# Causal inference in conservation policy: Evaluating the effects of Protected Areas on deforestation and well-being in the Peruvian Amazon

**DOI:** 10.1371/journal.pone.0337468

**Published:** 2026-10-14

**Authors:** Teresa L. Paredes, Karen I. Eckhardt

**Affiliations:** Department of Economics and Environmental Management, Universidad Antonio Ruiz de Montoya, Lima, Peru; Wallaga University, ETHIOPIA

## Abstract

The Peruvian Amazon, one of the most biodiverse regions on Earth, is under increasing pressure from deforestation, threatening both ecological wealth and local livelihoods. Protected Areas (PAs) have emerged as one of the main conservation strategies; however, questions remain regarding their effectiveness in reducing deforestation and their socioeconomic impact. This study evaluates the effects of PAs, their Buffer Zones (BZs), and Regional Conservation Areas (RCAs) on deforestation and household welfare between 2001 and 2023. Using causal inference methods, geographic Regression Discontinuity Design (RDD) and staggered Difference-in-Differences (DiD), the analysis integrates high-resolution spatial data on forest loss with georeferenced household information. Results indicate that PAs significantly reduce long-term deforestation by 1.3 to 2.8 ha/km^2^, while BZs mitigate forest loss by 1.1 to 1.5 ha/km^2^. However, no significant deforestation effects are observed in the short term. Regarding welfare, households within BZs exhibit lower per capita income and expenditure, particularly near indirect-use PAs. The study also provides exploratory evidence on RCAs, a category rarely examined in the literature. Although their estimated effects are smaller and not statistically significant, their inclusion highlights the growing role of regional governance in conservation policy. These findings indicate that conservation outcomes strengthen over time and depend on local governance, institutional capacity, and the ability to contain pressures from extractive activities. This research contributes new empirical evidence to inform conservation policy design, emphasizing the need to integrate ecological and social dimensions into Protected Area management.

## Introduction

Globally, forests cover more than 4 billion hectares and play important ecological and economic roles [1]. Peru ranks ninth worldwide in forest area, with over 72 million hectares, equivalent to 57.3% of its national territory [2]. These forests contribute around 1.04% of the national GDP, approximately USD 2,368 million, and provide ecosystem services that are essential to sustaining life on the planet [2]. One of the most important conservation policies to safeguard these services has been the establishment of Protected Areas.

In Peru, there are 77 nationally administered Protected Areas (PAs), 32 Regional Conservation Areas (RCAs), and 173 Private Conservation Areas, which together protect 17.88% of the country’s land surface [3]. These categories are regulated primarily by Law No. 26834 [4], which sets the primary objectives of PAs as conserving biodiversity, protecting habitats, and maintaining ecosystem services. PAs within the National System of State-Protected Natural Areas (SINANPE) are distinguished from complementary areas according to their governance regime, whether national, regional, or private, and the level of human intervention permitted. The regulation further differentiates between direct-use areas, where controlled activities compatible with conservation objectives are allowed, and indirect-use areas, where alteration of the natural environment is strictly prohibited.

Buffer Zones (BZ), defined in Article 25 of the law’s regulation, serve as transition areas surrounding PAs and aim to reduce the adverse effects of human activities. Permitted activities within BZs are specified in each PA’s Master Plan and must not compromise conservation objectives. Although BZs are not formally part of the PA, they play a strategic role in containing external pressures.

In recent years, Peru has made significant progress toward international conservation commitments. By 2020, the country achieved the Aichi target of protecting 17% of terrestrial areas under the Convention of Biological Diversity, with more than 22 million hectares designated as PAs [5]. More recently, Peru joined the Kunming-Montreal Global Biodiversity Framework adopted at COP15 and the 30x30 initiative, which aims to protect 30% of terrestrial and marine areas by 2030 [6].

The literature nonetheless cautions against evaluating these targets solely by the extent of protected land, since relying on numerical area coverage risks prioritizing quantity over quality. Arneth et al. [7] argue that ensuring meaningful outcomes for biodiversity, climate, and food security requires policy to move beyond simple area metrics and explicitly address management effectiveness. Visconti et al. [8] add that setting targets without robust criteria for management and ecological representativeness can incentivize so-called paper parks that fail to provide necessary connectivity or address real threats. Geldmann [9] further notes that achieving percentage-based goals, such as the 30x30, will be insufficient unless PAs are properly located and managed, since a narrow focus on area risks neglecting the socioeconomic dimensions essential for effective conservation.

Several studies question the real impact of PAs on these grounds, noting that they are often located in low-pressure zones, such as remote or unproductive lands, where low deforestation rates might have occurred even without formal protection. For example, Jusys [10] shows how location bias influences perceived deforestation avoidance across different protection types in the Brazilian Amazon. Similarly, Hernández et al. [11] emphasize that failing to account for spatial selectivity leads to an overestimation of conservation effectiveness; in Queensland, specifically, it was estimated that 89.5% of strictly protected PAs were unlikely to have been cleared even if they had never been protected. These findings raise the question of whether observed conservation outcomes are truly attributable to legal protection or merely reflect the inherent characteristics of the selected sites.

Evidence of effectiveness is mixed across regions. Significant reductions in deforestation have been documented in Latin America and Africa [12], while findings in Asia are more variable and in some cases inconclusive [13,14]. Large net forest losses have been recorded within PAs in Cambodia [13], and extensive forest loss and fragmentation have been observed in surrounding landscapes in Malaysia [14]. Governance regimes and management models are similarly contested. Community-based regimes in Africa decreased tree cover loss by 66% in high-pressure scenarios [15], whereas other research finds that stricter protection achieves better results under comparable pressure [12,16].

The role of BZs in maintaining forest cover and improving landscape connectivity has also been highlighted [17]. Yet, the possibility of deforestation leakage, where conservation efforts displace land-use activities to adjacent unprotected areas, remains under debate [18–21]. Ford et al. [20] found evidence of leakage in 46% of pan-tropical PAs, while Fuller et al. [21] estimate that positive spillover, sometimes termed blockage, is roughly five times more common globally. Institutional context also shapes policy performance, as governance factors such as policy stringency, property rights, and the rule of law help determine whether a forest policy succeeds in reducing tree-cover loss [22,23].

Although terminology and legal status vary across countries, BZs and RCAs share conceptual ground with categories studied elsewhere. BZs resemble the multiple-use or transition zones documented around strictly protected PAs in other regions, where permitted activities are more flexible than within the core area but remain subject to conservation objectives [17]. RCAs parallel the subnational or regionally governed reserves found in decentralized conservation systems, whose effectiveness has been linked to local governance capacity and proximity to communities [22,23].

From a methodological perspective, Grupp et al. [24] apply the staggered Difference-in-Differences model (DiD) proposed by Callaway and Sant’Anna [25] to evaluate the effect of PAs in Europe over three decades. Their findings suggest no significant impact on vegetation cover, possibly because the areas studied were located in low economic activity zones where natural recovery may have occurred regardless of protection.

In the Peruvian case, studies have primarily focused on the impacts of PAs on deforestation, often overlooking BZs and RCAs. Díaz y Miranda [26] and Miranda et al. [27] report significant reductions in deforestation associated with PA establishment. Orihuela and Pérez [28] find a similar pattern but additionally document limited or even negative effects on household welfare. Other studies highlight that effectiveness varies according to governance regimes and management models [29]. External pressures such as mining and agriculture are found to particularly affect BZs [30,31]. Additionally, most research has focused on PAs established prior to 2001 and has excluded those under regional administration, leaving an empirical gap addressed here by extending the temporal horizon and territorial scope of the analysis. Three factors explain why this gap persisted. First, evaluating PAs established after 2001 requires sufficient pre- and post-treatment data, a condition only met as the deforestation record extended further beyond their establishment dates, a condition not yet met in earlier studies covering periods through 2012 or 2016. Second, prior Peruvian studies relied primarily on matching methods, which do not exploit temporal variation and are therefore unsuited to estimating dynamic treatment effects for areas with staggered establishment dates. Third, Regional Conservation Areas, governed at the subnational level outside the SINANPE framework, have been consistently overlooked in the empirical literature, leaving their conservation performance largely unexamined despite their growing role in Amazonian governance [26].

While deforestation is an important indicator of conservation outcomes, it is not the sole dimension of PA effectiveness [29]. The social impacts of PAs are similarly heterogeneous in the literature. PAs can generate income through compatible activities like ecotourism and community-based enterprises [32–34], while restrictions on resource access can produce adverse welfare effects if not accompanied by viable alternatives [35,36]. Living near a PA does not guarantee welfare improvements and may instead create costs, such as crop damage from wildlife or uneven distribution of tourism benefits [37,38]. Kandel et al. [39] identify a moderate positive effect on income overall, with larger impacts observed in Asia and Latin America.

Conservation policies therefore need an integrated approach that anticipates and mitigates negative externalities, above all those arising from restrictions on resource access and use by local populations [34,40,41]. In Peru, empirical work linking environmental impacts and household welfare, especially from BZs and RCAs, remains limited. A growing body of literature emphasizes that PAs’ effects are not static but evolve over time, which calls for designs that explicitly account for temporal dynamics [42–44].

To address these gaps, the present work jointly evaluates environmental and social outcomes of PAs, BZs, and RCAs in the Peruvian Amazon. We exploit two complementary causal inference strategies to capture both long-term, accumulated effects and shorter-term, dynamic responses. The distinction between these two temporal horizons is driven by data availability, since high-resolution, annually disaggregated deforestation data are available only from 2001 onward. This defines the threshold separating areas whose cumulative protection effects can be measured cross-sectionally, established before 2001, from those for which pre- and post-treatment panel data exist, established after 2001. For the former, a geographical Regression Discontinuity Design (RDD) leverages legal boundaries to estimate local average treatment effects for units adjacent to the border. For the latter, the staggered DiD framework of Callaway and Sant’Anna [25] is applied to estimate cohort- and event-time-specific effects while avoiding biases arising from already-treated units serving as controls. A detailed justification of the temporal threshold is provided in the Empirical Strategy section. From a causal inference perspective, the analysis frames legal protection as the treatment and aims to estimate its attributable effect on deforestation and household welfare relative to a constructed counterfactual scenario representing what would have occurred without protection [45].

Although impact evaluations of PAs have been conducted in tropical regions, the methodological combination, governance classifications, and temporal coverage employed here are specific to the Peruvian context. To our knowledge, this is the first study to apply a staggered DiD to the analysis of PAs in Peru, while simultaneously evaluating three distinct governance categories, nationally administered PAs, BZs, and RCAs, within a single causal inference framework, and over the longest temporal horizon available in the national literature. By integrating ecological and socio-economic outcomes under a unified identification strategy, the findings aim to inform conservation policy design in ways that balance environmental effectiveness with social equity.

The paper is organized as follows: the Materials and Methods section describes the data and the RDD and staggered DiD models; the Results section reports the main findings; the Discussion section interprets them and outlines limitations and policy implications; and the Conclusion section summarizes the study.

## Materials and methods

### Study area

This study focuses on the Peruvian Amazon, which covers nearly 60% of the national territory and concentrates most of the country’s forest resources [2]. Two units of observation were employed. For the deforestation analysis, the unit is a 1 km^2^ grid cell overlaid on the Amazon forest. Although the source deforestation data from MINAM are available at 30 m pixel resolution, a 1 km^2^ grid was used given the computational demands of processing the full Amazonian coverage at finer granularity, consistent with previous studies in similar contexts [28,46]. Following Orihuela and Perez [28], a cell is classified as treated if the PA or BZ boundary intersects it, and as control otherwise. The distance to the boundary is assigned as negative for treated cells and positive for control cells. For the welfare analysis, the unit is the household, using georeferenced information from the National Household Survey (ENAHO).

Treatment and control units are assigned according to their location within three zones: inside the PA (Zone 1), inside its BZ (Zone 2), and outside both (Zone 3) [28] (Fig 1). To estimate the effect of PAs, units located within the Protected Area are compared to those in the Buffer Zone. For BZs and RCAs, which follow the same assignment criteria, treated units correspond to those located within the respective area, while control units are those situated outside.

**Fig 1:**
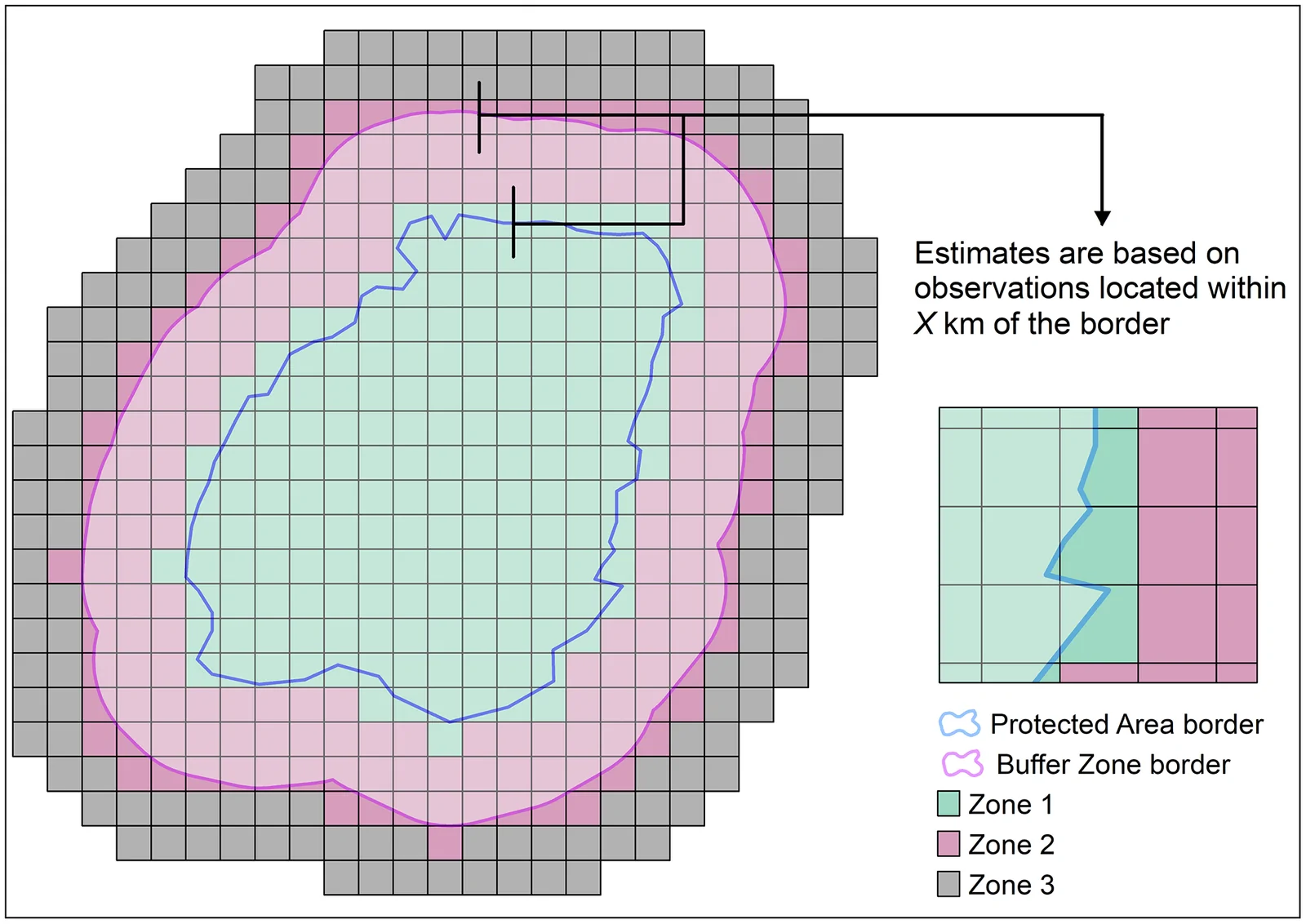
Schematic representation of treatment and control unit assignment based on area type. Zone 1: inside the PA (treated), Zone 2: inside the BZ (treated) Zone 3: outside both (control). PA effects are estimated comparing Zone 1 to Zone 2; BZ and RCA effects compare Zone 2 or the RCA interior to Zone 3. The bandwidth indicates the distance window used for estimation. PA: Protected Area, BZ: Buffer Zone; RCA: Regional Conservation Area. Sources: Authors’ elaboration.

To provide a clearer understanding of the analytical sample, Fig 2 illustrates the spatial distribution of all PAs across the Peruvian Amazon. The figure distinguishes between those incorporated into the short-term and long-term analyses and those excluded due to data constraints or the timing of their legal establishment.

**Fig 2:**
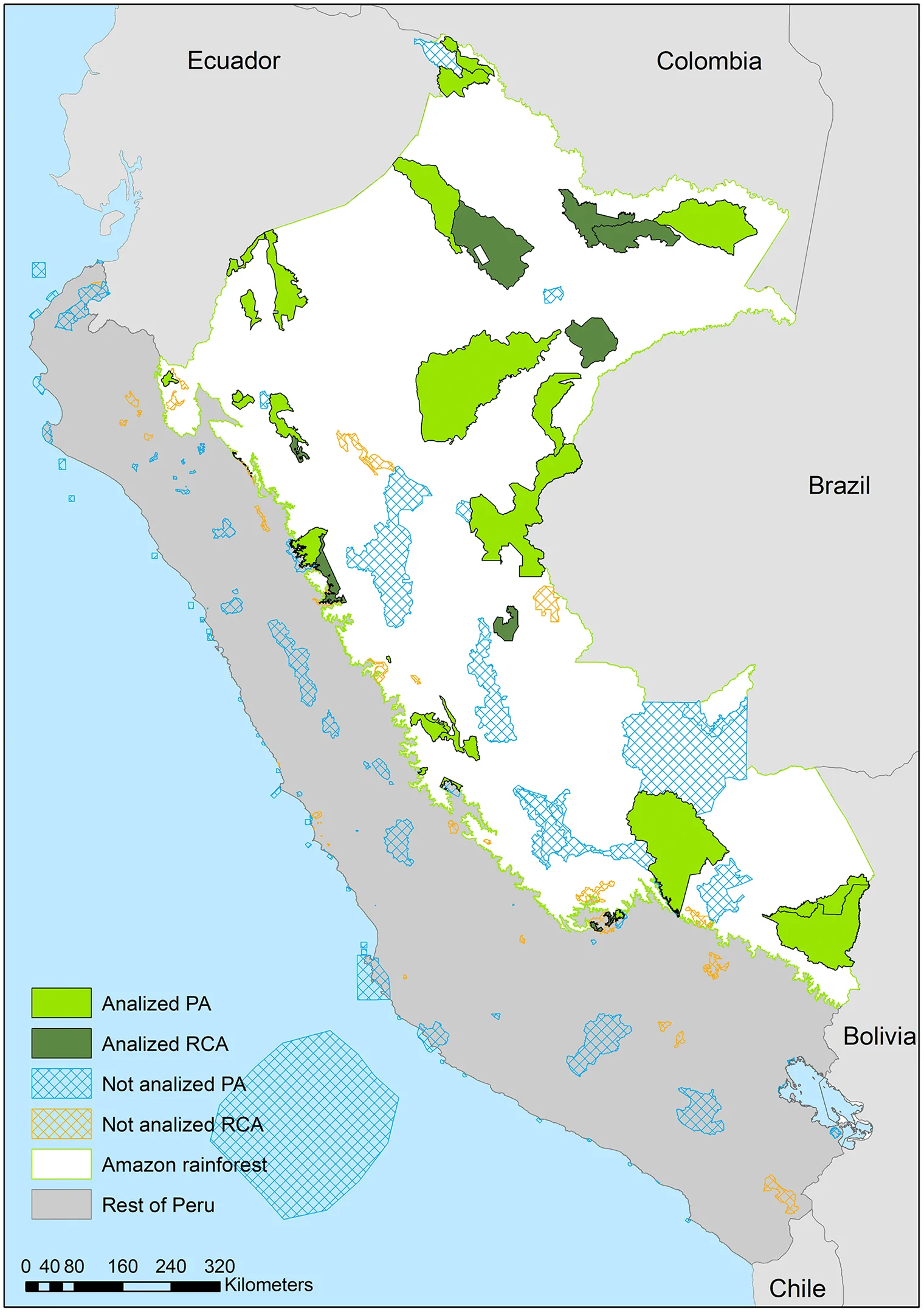
Protected Areas in the Peruvian Amazon included in the analysis. Areas established before 2001 enter the long-term RDD analysis. Areas established between 2006 and 2018 enter the short-term staggered DiD analysis. Sources: Authors’ elaboration based on geospatial data from MINAM and SERNANP, used with permission (see S1 Table for details).

In some cases, the GIS-based calculations of PA areas derived from spatial processing did not perfectly match the official legal areas reported by SERNANP. This discrepancy, also documented in previous studies [47], arises from differences between legal boundaries and spatial datasets. For consistency with the geospatial analysis, the GIS-based areas were used in this study.

To avoid potential bias, observations that overlap with other conservation categories are excluded. In cases where two PAs share a boundary, each unit is assigned to the nearest area. Reserved Zones are included only in aggregate models, given their transitional status, which precludes meaningful disaggregation by category.

### Data sources

The main outcomes are deforestation and household welfare. Forest loss data were obtained from the Ministry of Environment (MINAM) through the Programa Nacional de Conservación de Bosques, available for 2001–2023 at 30-m resolution. Welfare outcomes are measured with household income and expenditure per capita, using ENAHO survey modules on housing characteristics, education, and expenditure aggregates.

Additional controls are included to account for geographic and accessibility factors that may independently influence deforestation or welfare outcomes. Physical terrain controls comprise altitude, slope, temperature, and precipitation, which capture natural conditions shaping both forest cover and land-use pressure. Soil quality, derived from the FAO Harmonized World Soil Database, proxies agricultural suitability. Accessibility variables measure distance to roads, rivers, mining concessions, forest concessions, oil blocks, and native communities, capturing the intensity of human pressure and economic activity near PA, RCA, and BZ boundaries; these were obtained from official government sources (MTC, INGEMMET, SERFOR, Perupetro, IBC, SERNANP). For the welfare analysis, household-level controls include sex, age, education level, and household size, drawn from ENAHO modules 1, 3, and 34. A detailed description of all variables, including definitions, units of measurement, data sources, and the analysis in which each is used, is presented in S1 Table.

### Empirical strategy

The empirical strategy relies on two causal inference methods, considering that the assignment of legal protection is not random [45]. Causal inference is understood here as the estimation of the effect attributable to an intervention, specifically, the establishment of a PA, by comparing the observed outcome with a counterfactual scenario in which protection did not occur.

Two approaches are employed according to the temporal availability of data. For long-term analysis, a Regression Discontinuity Design (RDD) was implemented, following the approach of previous studies conducted in Colombia [48,49] and Peru [28]. For short-term analysis, a staggered Difference-in-Differences (DiD) model was used, based on the framework proposed by Grupp et al. [24].

### Long-term analysis: Regression Discontinuity Design (RDD)

For PAs and BZs established before 2001, a sharp RDD was applied to a total of 14 PAs within the study area.

The assignment variable can be interpreted in one or two dimensions. In one dimension, it corresponds to the Euclidean distance from the boundary of the PA: a negative value indicates that the observation is located inside the area (treatment), and a positive value indicates it is outside (control). In two dimensions, the discontinuity is evaluated using the geographic coordinates (latitude and longitude) of each observation. The model was estimated using first- and second-degree polynomial terms in latitude and longitude. The use of distance to the boundary as the running variable is appropriate in this setting because the legal limits of PAs and BZs were not drawn based on proximity to populated areas or pre-existing land-use intensity. Rather, they reflect conservation priorities and ecological criteria established through national legislation and institutional processes [4,29]. This means that the cutoff, the boundary itself, is plausibly exogenous to the socioeconomic and deforestation outcomes of interest, a necessary condition for the RDD to produce credible causal estimates [50].

The RDD model relies on the assumption that units situated near the boundary of the PA are comparable in both observable and unobservable characteristics [50]. Under this assumption, the estimated effect is locally valid and can be interpreted as the causal impact for observations near the boundary, assuming that covariates vary continuously across it. This assumption is plausible in the present context for several reasons. First, PAs and BZs boundaries in Peru are defined through legal and administrative instruments, primarily Law N° 26834 and individual Master Plans, rather than following pre-existing socioeconomic gradients or deforestation pressure levels. As a result, households and grid cells on either side of these boundaries are unlikely to differ systematically in unobservable characteristics solely because of their position relative to the limit. Second, the model includes a comprehensive set of geographic and accessibility controls, which account for the observable gradients most likely to confound the estimates. Third, boundary fixed effects are included to capture any time-invariant, area-specific unobservable that might vary systematically across different PAs and BZs. Finally, this identification strategy closely follows previous applications of geographic RDD to PAs in Colombia [48,49] and Peru [28], where the same assumption has been argued to hold on the basis of the administrative nature of the boundaries. The validity of these assumptions is further corroborated by the robustness checks presented in the Results section.

As a preliminary step, a local conditional means test was conducted to assess the suitability of the RDD framework. The test compared average values of deforestation, income, and expenditure using equidistant bins around PAs and BZs boundaries.

Having established the plausibility of the identifying assumptions, the estimated model is specified as follows (Equation 1):


$$\begin{array}{c} Y_{ia}=\beta_{\text{0}}+\beta_{\text{1}}T_{i}+g\left(D_{i}\right)+\beta_{\text{2}}X_{i}+\varphi_{i}+\mu_{i} \end{array}$$ (1)

The dependent variable $Y_{ia}$ represents one of the following outcomes for observation *i*, at boundary *a*: (i) hectares deforested per square kilometer, or (ii) real monthly per capita household income or expenditure divided by the poverty line. The treatment variable $T_{i}$ takes the value of 1 if the unit of analysis (grid cell or household) is located inside the PA or BZ, and 0 if it is outside. The parameter $\beta_{\text{1}}$ captures the causal impact of PA or BZ designation on deforestation or welfare. The function $g\left(D_{i}\right)$ is a polynomial in the distance to the boundary, in one or two dimensions depending on the specification. The vector $X_{i}$ includes two sets of controls depending on the outcome of interest. For the deforestation analysis, controls capture physical and accessibility conditions, comprising terrain characteristics such as altitude, slope, temperature, precipitation, and soil quality, as well as distance to roads, rivers, mining concessions, forest concessions, oil blocks, and native communities, all of which proxy the land-use pressures most likely to confound the boundary comparison. For the welfare analysis, controls include socioeconomic characteristics of the household head, namely sex, age, education level, and household size, as described in the Data sources section and detailed in S1 Table. $\varphi_{i}$ represents fixed effects by boundary, and $\mu_{i}$ is the error term, clustered at the 10-km grid level [28,46].

Due to the limited number of households observed within PAs, the welfare analysis is restricted to BZs only.

### Short-term analysis: Staggered Difference-in-Differences (DiD)

For PAs, BZs, and RCAs established after 2001, the staggered Difference-in-Differences (DiD) model developed by Callaway and Sant’Anna [25] was applied. This approach addresses a limitation of the classical DiD framework, which arises from using already treated units as controls in later periods. The methodology is particularly well suited to contexts in which treatment adoption is staggered over time, enabling the identification of dynamic effects [24,51,52].

The model relies on the parallel trends assumption, which states that in the absence of treatment, differences between treated and control groups would remain constant over time. Under this assumption, the Average Treatment Effect on the Treated is estimated as the difference in temporal changes between treated and control groups [53].

Callaway and Sant’Anna [25] propose three estimators to express the causal impact of treatment: (i) effects by time since exposure (event time), (ii) cumulative effects up to a specific period (event calendar), and (iii) effects by treatment adoption period (cohort). The analysis was conducted for a total of 11 PAs and 9 RCAs. The RCA sample is smaller than the PA sample, a consequence of the relatively recent establishment of most RCAs in Peru and the more stringent temporal criteria required for the DiD design. Given the limited sample size, the RCA analysis is regarded as exploratory. Accordingly, estimated effects should be interpreted with caution, as the statistical power to detect moderate conservation effects is lower for the RCAs than for the PAs.

The model is specified as follows (Equation 2):


$$\begin{array}{c} Y=\alpha_{\text{1}}^{g,t}+\alpha_{\text{2}}^{g,t}*G_{g}+\alpha_{\text{3}}^{g,t}*\text{1}\left\{T=t\right\}+\beta^{g,\;t}*\left(G_{g}*\text{1}\left\{T=t\right\}\right)+\gamma X+\varepsilon^{g,t} \end{array}$$ (2)

The outcome of interest *Y* is deforestation in hectares per square kilometer for grid cell *i* at time *t*. The treatment variable is defined by the year *G* in which the surface containing the grid cell becomes part of a PA, BZ, or RCA. Its evolution is compared to that of grid cells located in areas not yet protected or never protected. A vector of controls *X* is included, comprising the same terrain and accessibility variables used in the RDD deforestation analysis, given the shared unit of observation. The welfare analysis is not extended to the DiD framework. Errors are clustered at the 10 km^2^ grid level. The coefficient of interest $\beta^{g,t}$ represents the average causal effect of PA, BZ, or RCA designation on deforestation for cohort *g* in year *t* after treatment.

The distinction between long-term and short-term analysis in this study does not arise from the methods themselves, but from data availability and the nature of the intervention. High-resolution deforestation data are available only from 2001 onward, which makes it impossible to observe pre-treatment trends for PAs established before that year and therefore precludes a panel-based evaluation for those areas. The label *long-term* reflects the fact that, by 2023, PAs in the RDD sample had been under legal protection for a minimum of 22 years, allowing the RDD to capture their accumulated conservation impact through spatial variation at legal boundaries. Conversely, for areas established after 2001, the data window provides sufficient pre- and post-treatment observations to implement a credible DiD design. Within the short-term sample, the analysis is restricted to areas established between 2006 and 2018. The lower bound ensures a minimum of five pre-treatment years, necessary to test the parallel trends assumption, while the upper bound guarantees at least five post-treatment years of observation, consistent with evidence that conservation effects require time to materialize [43,44]. This temporal distinction does not imply that the RDD is inherently a long-term method or the DiD a short-term one. Both are adapted here to maximize the use of available information given the institutional and data constraints of the study setting.

## Results

### Long-term analysis: Regression Discontinuity Design (RDD)

Fig 3 represents the results of the local conditional means test conducted to assess the suitability of the RDD framework. The X-axis indicates the distance to the boundary, where zero corresponds to the legal limit of the PA or BZ; negative values denote treated units located inside, while positive values represent control units outside. The Y-axis shows the average value of the outcome variable in each bin. The discontinuities observed confirm that the identifying assumptions hold. Deforestation declines sharply when crossing from BZs into PAs (Fig 3A) and, though less pronounced, also when entering BZs from surrounding areas (Fig 3B). Conversely, income and expenditure decrease within BZs compared with adjacent areas (Fig 3C and 3D).

**Fig 3:**
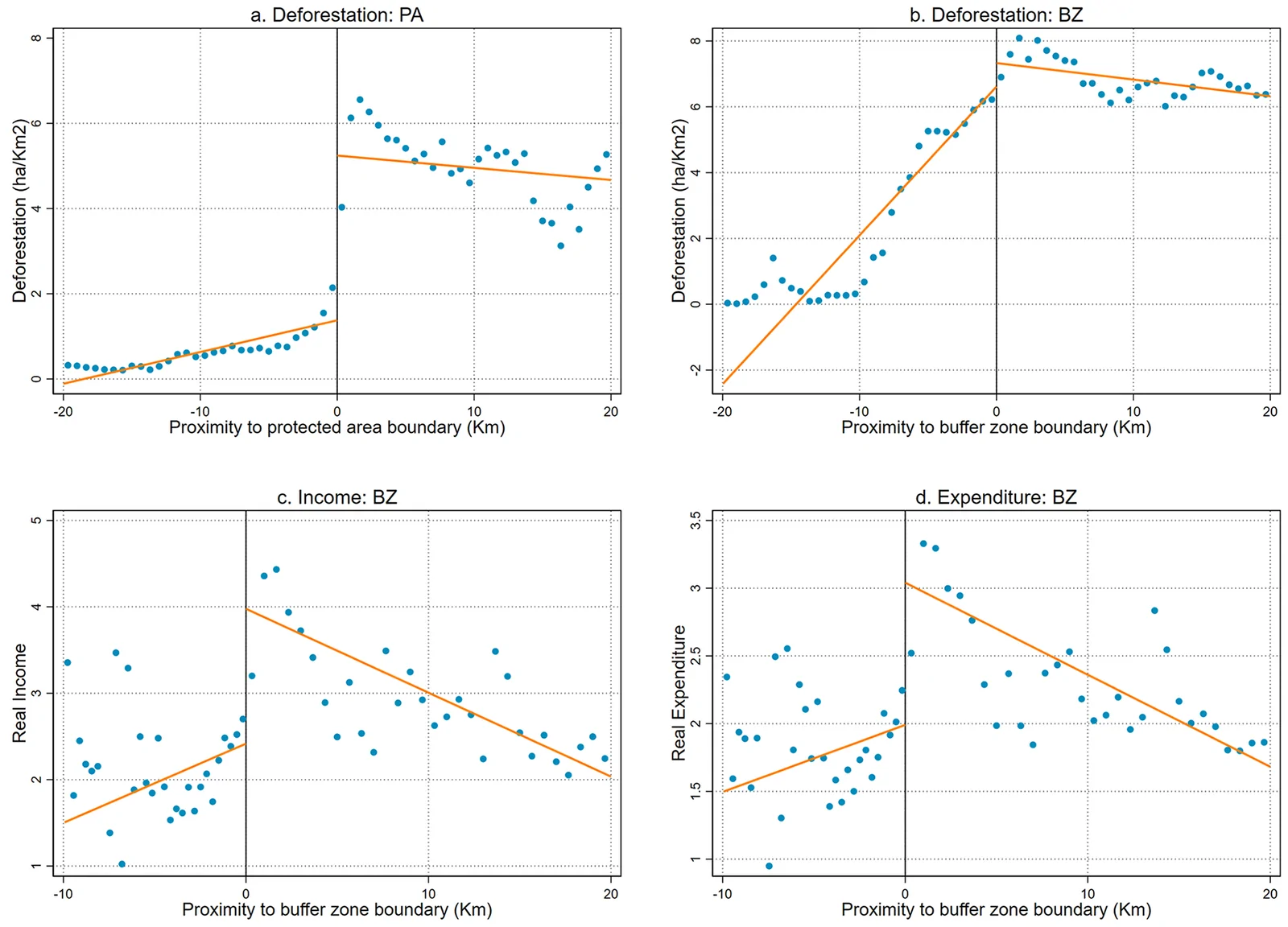
Local conditional means of deforestation and welfare outcomes by distance to PA and BZ boundaries. Each point represents the mean outcome within equidistant 1 km bins. Negative values: treated units (inside PA or BZ); positive values: control units (outside). Zero marks the legal boundary. (a) Deforestation near PA boundaries (ha/km^2^); (b) Deforestation near BZ boundaries (ha/km^2^); (c) Household income near BZ boundaries (expressed as a ratio to the poverty line); (d) Household expenditure near BZ boundaries (expressed as a ratio to the poverty line).

Table 1 reports the estimated causal effects for PAs and BZs established before 2001. When using two-dimensional coordinates (latitude and longitude) as the running variable, deforestation declines by 2.2 to 3.5 ha/km^2^ inside PAs (Table 1, Panel A) and by 0.8 to1.2 ha/km^2^ inside BZs. When using linear distance to the boundary as the running variable, reductions range from 1.3 to 2.8 ha/km^2^ for PAs and 1.1 to 1.5 ha/km^2^ for BZs (Table 1, Panel C). These results are robust across model specifications, inclusion of controls, and alternative bandwidths.

**Table 1:** Causal effects of Protected Areas and Buffer Zones on cumulative deforestation, by specification and bandwidth.

	PA			BZ		
	< 1 km of boundary	< 5 km of boundary	< 10 km of boundary	< 1 km of boundary	< 5 km of boundary	< 10 km of boundary
Panel A. Third-order Polynomial in Latitude and Longitude						
Treatment	−2.227***	−3.358***	−3.542***	−1.174***	−1.133***	−0.849**
	(0.325)	(0.388)	(0.390)	(0.333)	(0.387)	(0.367)
*R*^2^	0.164	0.194	0.192	0.308	0.320	0.312
Panel B. Second-order Polynomial in Distance to the Boundary						
Treatment	−1.434***	−2.486***	−2.932***	−1.078***	−1.494***	−1.123**
	(0.400)	(0.430)	(0.474)	(0.363)	(0.402)	(0.466)
*R* ^2^	0.150	0.192	0.188	0.289	0.302	0.297
Panel C. First-order Polynomial in Distance to the Boundary						
Treatment	−1.265***	−2.183***	−2.776***	−1.481***	−1.447***	−1.113**
	(0.323)	(0.393)	(0.410)	(0.333)	(0.405)	(0.467)
*R*^2^	0.150	0.191	0.187	0.289	0.302	0.295
Obs.	13,159	36,689	55,643	14,228	41,444	62,249

Geographic RDD estimates. Dependent variable: cumulative deforestation (ha/km^2^, 2001–2023). Columns: first-, second-, and third-degree polynomial specifications, with geographic controls. Rows: ± 1 km, ± 5 km, and ± 10 km bandwidths. Boundary fixed effects are included, and standard errors are adjusted for clustering at the 10 km^2^grid level. Robust standard errors in parentheses. *** p < 0.01, ** p < 0.05, * p < 0.1.

Heterogeneity emerges across PA categories. Communal Reserves and Protection Forests show the strongest impacts, with reductions up to 7.5 ha/km^2^ and 4.2 ha/km^2^, respectively, while National Sanctuaries and Reserved Zones exhibit no significant effects (Table 2).

**Table 2:** Heterogeneous effects of Protected Areas on deforestation by conservation category and bandwidth.

	Historic Sanctuary	Protection Forest	National Park	Communal Reserve	National Reserve	National Sanctuary	Reserved Zone
< 1 km of boundary							
PA	−1.717**	−1.890*	−1.235***	−3.835***	−0.859**	−0.335	−0.076
	(0.378)	(0.981)	(0.458)	(1.202)	(0.430)	(0.878)	(0.326)
*R*^2^	0.371	0.221	0.196	0.309	0.173	0.295	0.081
Obs.	117	2,597	4,380	318	3,919	216	1,612
< 5 km of boundary							
PA	−1.579***	−3.049**	−2.107***	−5.681***	−1.597**	−0.853	−0.304
	(0.434)	(1.180)	(0.610)	(1.124)	(0.618)	(0.649)	(0.323)
*R*^2^	0.281	0.257	0.204	0.411	0.205	0.328	0.121
Obs.	284	6,320	12,405	812	12,016	490	4,362
< 10 km of boundary							
PA	−2.028***	−4.195***	−2.504***	−7.450***	−2.340***	−0.772	−0.401
	(0.611)	(1.236)	(0.680)	(0.833)	(0.660)	(0.655)	(0.309)
*R*^2^	0.135	0.256	0.189	0.464	0.227	0.395	0.162
Obs.	489	7,320	19,981	1,067	20,475	499	5,812

Geographic RDD estimates by PA category: Historic Sanctuaries, Protection Forests, National Parks, Communal Reserves, National Reserves, National Sanctuaries, and Reserved Zones. Dependent variable: cumulative deforestation (ha/km^2^, 2001–2023). First-degree polynomial with geographic controls. Rows report estimates for observations located within <1 km, < 5 km, < 10 km of the boundary. Boundary fixed effects are included, and standard errors are adjusted for clustering at the 10 km^2^ grid level. Robust standard errors in parentheses. *** p < 0.01, ** p < 0.05, * p < 0.1.

The performance of Buffer Zones also varies. Those associated with Communal Reserves reduce deforestation by 6.7–9.7 ha/km^2^, whereas effects are weaker or absent in most other categories (Table 3).

**Table 3:** Heterogeneous effects of Buffer Zones on deforestation by associated protected area category and bandwidth.

	Historic Sanctuary	Protection Forest	National Park	Communal Reserve	National Reserve	National Sanctuary	Reserved Zone
< 1 km of boundary							
BZ	−0.004	−1.525*	−2.419***	−8.054***	−0.946*	2.280**	0.485
	(1.352)	(0.878)	(0.606)	(2.381)	(0.484)	(0.924)	(0.558)
*R*^2^	0.183	0.297	0.285	0.191	0.399	0.477	0.195
Obs.	173	2,734	4,213	338	4,994	179	1,597
< 5 km of boundary							
BZ	−1.248*	−2.855***	−1.363	−9.728***	−0.168	1.536	0.224
	(0.703)	(1.065)	(0.826)	(3.004)	(0.551)	(1.206)	(0.429)
*R*^2^	0.183	0.324	0.236	0.175	0.389	0.377	0.153
Obs.	722	6,406	12,776	650	16,504	457	3,929
< 10 km of boundary							
BZ	−1.340*	−4.242***	−0.603	−6.663*	−0.052	0.774	0.772*
	(0.732)	(1.400)	(0.840)	(3.194)	(0.697)	(1.096)	(0.391)
*R*^2^	0.138	0.315	0.220	0.166	0.372	0.213	0.128
Obs.	1,169	9,551	19,800	747	24,497	816	5,669

Geographic RDD estimates for BZs stratified by associated PA category. Dependent variable: cumulative deforestation (ha/km^2^, 2001–2023). First-degree polynomial with geographic controls. Rows report estimates for observations located within <1 km, < 5 km, and <10 km of the boundary. Boundary fixed effects are included, and standard errors are adjusted for clustering at the 10 km^2^ grid level. Robust standard errors in parentheses. *** p < 0.01, ** p < 0.05, * p < 0.1.

Differences by use type are also notable. Direct-use PAs achieve larger reductions in deforestation (1.5–3.1 ha/km^2^) than indirect-use PAs (1.2–2.4 ha/km^2^). However, the pattern is reversed in buffer zones, Where those surrounding indirect-use areas reduce deforestation by up to 2.2 ha/km^2^, compared with 1.6 ha/km^2^ in direct-use zones (Table 4).

**Table 4:** Effects of Protected Areas and Buffer Zones on deforestation by permitted use type and bandwidth.

	PA		BZ	
	Indirect-use	Direct-use	Indirect-use	Direct-use
< 1 km of boundary				
Treatment	−1.199***	−1.468***	−2.166***	−−1.594***
	(0.441)	(0.461)	(0.573)	(0.466)
*R*^2^	0.181	0.171	0.279	0.328
Obs.	4,713	6,834	4,565	8,066
< 5 km of boundary				
Treatment	−2.052***	−2.375***	−1.288*	−1.462***
	(0.570)	(0.555)	(0.767)	(0.548)
*R*^2^	0.196	0.227	0.229	0.353
Obs.	13,179	19,148	13,955	23,560
< 10 km of boundary				
Treatment	−2.446***	−3.149***	−0.591	−1.408**
	(0.636)	(0.580)	(0.779)	(0.663)
*R*^2^	0.184	0.234	0.213	0.345
Obs.	20,969	28,862	21,785	34,795

Geographic RDD estimates stratified by use type: direct-use (compatible activities permitted) and indirect-use (strict protection). Dependent variable: cumulative deforestation (ha/km^2^, 2001–2023). First-degree polynomial with geographic controls. Rows report estimates for observations located within <1 km, < 5 km, and <10 km of the boundary. Boundary fixed effects are included, and standard errors are adjusted for clustering at the 10 km^2^ grid level. Robust standard errors in parentheses. *** p < 0.01, ** p < 0.05, * p < 0.1.

Several cases stand out in the heterogeneous effects analysis. Tingo María National Park, despite its small size, shows large and consistent reductions (5.7–9 ha/km^2^), while Tambopata National Reserve exhibits reductions of 4.1–9.2 ha/km^2^. By contrast, extensive areas such as Pacaya Samiria and Manu National Park show no significant impacts, likely reflecting limited external pressures. Significant containment effects are also identified in the BZs of Alto Mayo Protection Forest and Yanesha Communal Reserve (see S2 and S3 Tables).

Regarding welfare, households located within BZs report lower income and expenditure relative to those outside. On average, income decreases by 0.05–0.45 times the poverty line and expenditure by 0.04–0.28 times the poverty line (Table 5).

**Table 5:** Effects of Buffer Zones on household income and expenditure by specification and bandwidth.

	Income			Expenditure		
	< 1 km of boundary	< 5 km of boundary	< 10 km of boundary	< 1 km of boundary	< 5 km of boundary	< 10 km of boundary
Panel A. Third-order Polynomial in Latitude and Longitude						
BZ	−0.045	−0.231	−0.153	−0.035	−0.082	−0.058
	(0.163)	(0.162)	(0.130)	(0.079)	(0.076)	(0.069)
*R*^2^	0.208	0.172	0.158	0.338	0.355	0.350
Panel B. Second-order Polynomial in Distance to the Boundary						
BZ	−0.289	−0.362*	−0.194	−0.137	−0.223**	−0.158**
	(0.475)	(0.207)	(0.166)	(0.229)	(0.098)	(0.080)
*R*^2^	0.201	0.171	0.156	0.326	0.353	0.347
Panel C. First-order Polynomial in Distance to the Boundary						
BZ	−0.273	−0.445**	−0.198	−0.129	−0.282**	−0.169**
	(0.484)	(0.212)	(0.171)	(0.229)	(0.111)	(0.078)
*R*^2^	0.200	0.170	0.156	0.325	0.352	0.347
Obs.	4,858	16,872	21,395	4,858	16,872	21,395

Geographic RDD estimates using georeferenced ENAHO data (2007–2023). Dependent variables: real monthly per capita household income and expenditure, (expressed as a ratio to the poverty line. Income and expenditure are deflated to constant 2021 soles using an annual CPI series, converted to monthly per capita terms, and then divided by the corresponding poverty line, following Orihuela and Pérez [28]. Panels report first-, second-, and third-degree polynomial specifications with socioeconomic controls; columns report estimates for observations located within <1 km, < 5 km, and <10 km of the boundary. Standard errors are clustered at the 10 km^2^ grid level and reported in parentheses. *** p < 0.01, ** p < 0.05, * p < 0.1.

The negative effect is more pronounced in BZs adjacent to indirect-use PAs, where monthly per capita income and expenditure fall by up to 1.2 and 0.6 times the poverty line, respectively, compared with 0.4 and 0.3 times the poverty line in BZs surrounding direct-use areas (Table 6).

**Table 6:** Effects of Buffer Zones on household welfare by associated Protected Area use type.

	Income		Expenditure	
	Indirect-use	Direct-use	Indirect-use	Direct-use
< 1 km of boundary				
BZ	−1.229**	0.395	−0.648***	0.250
	(0.494)	(0.525)	(0.184)	(0.267)
*R*^2^	0.142	0.248	0.242	0.365
*Obs.*	1,901	2,957	1,901	2,957
< 5 km of boundary				
BZ	−0.846**	−0.284	−0.321**	−0.290**
	(0.374)	(0.218)	(0.155)	(0.143)
*R*^2^	0.110	0.194	0.260	0.374
*Obs.*	4,562	12,310	4,562	12,310
< 10 km of boundary				
BZ	−0.415	−0.100	−0.180	−0.177*
	(0.376)	(0.155)	(0.143)	(0.092)
*R*^2^	0.098	0.187	0.267	0.373
*Obs.*	6,208	15,187	6,208	15,187

Geographic RDD estimates stratified by associated PA use type (direct-use vs. indirect-use). Dependent variables: real monthly per capita household income and expenditure, expressed as a ratio to the poverty line, using the same deflation and normalization procedure as in Table 5. ENAHO data (2007–2023). First-degree polynomial specification with socioeconomic controls; columns report estimates for observations located within <1 km, < 5 km, and <10 km of the boundary. Standard errors are clustered at the 10 km^2^ grid level and reported in parentheses. *** p < 0.01, ** p < 0.05, * p < 0.1.

For the main specification of the RDD model, which uses linear distance to the boundary of the PA or BZ as the assignment variable, three robustness checks were conducted. First, a donut-type RDD was applied by excluding observations closest to the boundary, ensuring that results are not driven by treatment assignment immediately adjacent to legal boundaries, which often do not coincide with physical barriers. Second, placebo tests were conducted at the boundaries by estimating the model using alternative bandwidths, confirming that the estimators are not sensitive to bandwidth choice. Third, artificial boundaries were introduced by shifting the actual boundary inward or outward, generating limits where no real treatment change occurs; under valid identification, no significant effects should be detected. In all cases, the results remain consistent in magnitude, direction, and statistical significance, thereby supporting the robustness of the estimates (see S1 Text). Taken together, these tests provide direct empirical support for the key identifying assumption of the RDD, that observable and unobservable covariates do not change discontinuously at the PA or BZ boundary. The absence of significant effects in placebo tests, where the boundary is shifted inward or outward to locations where no true treatment change occurs, confirms that the discontinuities detected in the main estimates are not artifacts of the spatial structure of the data. Similarly, the stability of the estimates across alternative bandwidths rules out that the results are driven by the particular choice of bandwidth. This supports the conclusion that units on either side of the legal boundary are locally comparable.

### Short-term analysis: Staggered Difference-in-Differences (DiD)

The short-term analysis relies on the staggered Difference-in-Differences model proposed by Callaway and Sant’Anna [25]. Estimates were conducted for PAs, BZs, and RCAs established between 2006 and 2018, ensuring at least five pre-treatment and five post-treatment years. The lower bound of 2006 corresponds to the minimum pre-treatment window required to reliably test the parallel trends assumption. Areas established between 2001 and 2005 are excluded because the available pre-treatment period within the data window (2001–2023) is insufficient for this purpose. The upper bound of 2018 ensures at least five post-treatment years of observations, which prior literature identifies is necessary for conservation effects to begin materializing [43,44].

Treatment and control assignment follows the same criteria as in the long-term analysis: grid cells located inside a PA, BZ, or RCA are assigned to the treatment group, while cells outside these areas (never treated) serve as controls. To avoid contamination, pixels located within PAs created before 2001 were excluded. The main specification relies on a ± 5-km bandwidth around boundaries.

As part of the preliminary analysis, the parallel trends assumption was tested for PAs, BZs, and RCAs by examining pre-treatment trends in deforestation for each cohort. Under this assumption, treated and control grid cells should follow similar deforestation trajectories before the establishment of the PAs. A joint test of pre-treatment coefficients showed no statistically significant violations for any of the three categories (p-values > 0.05 across all cohorts), supporting the validity of the design.

For PAs, no statistically significant reductions in deforestation are observed across the event-time, event-calendar, or cohort estimators (Fig 4). The trajectory indicates fluctuating but non-significant effects in the years following establishment (Fig 4A), consistent with the idea that conservation impacts require time to materialize.

**Fig 4:**
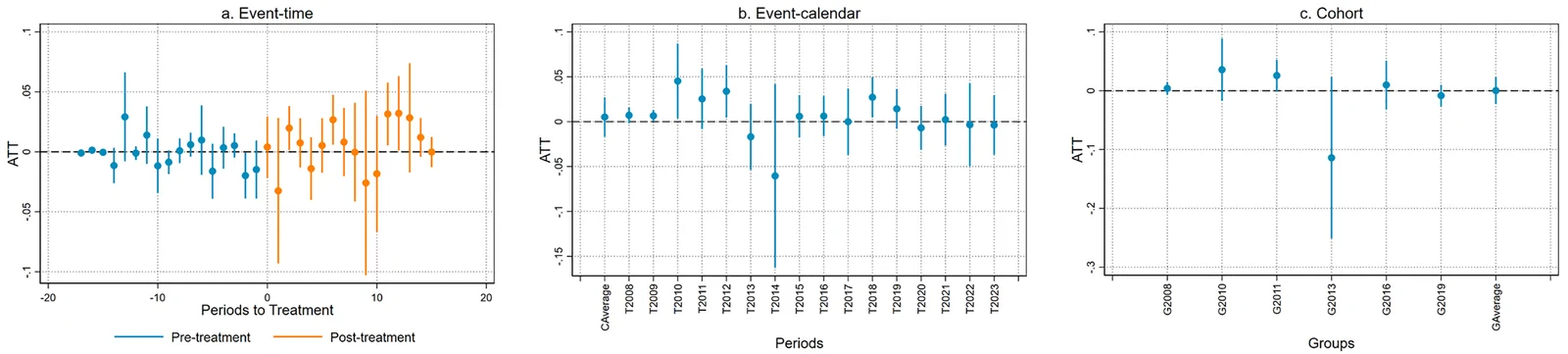
Short-term effects of Protected Area establishment across estimators. Staggered DiD estimates (Callaway and Sant’Anna [25]) for PAs established 2006-2018. (a) Event-time estimates; (b) Calendar-time estimates; (c) Cohort-level estimates. Outcome: annual deforestation (ha/km^2^). Bandwidth ± 5 km. Bars indicate 95% confidence intervals.

BZs show a more stable pattern, with sustained reductions in deforestation that peak in years 8 and 9, followed by a non-significant increase after year 14 (Fig 5A). BZs of PAs created between 2006 and 2018 show a maximum cumulative reduction of 0.119 ha/km^2^ by 2016 and an average reduction of 0.033 ha/km^2^ (Fig 5B). Cohort-level disaggregation reveals that BZs of PAs established in 2012 exhibit the largest cumulative reduction in deforestation, reaching 0.111 ha/km^2^ (Fig 5C).

**Fig 5:**
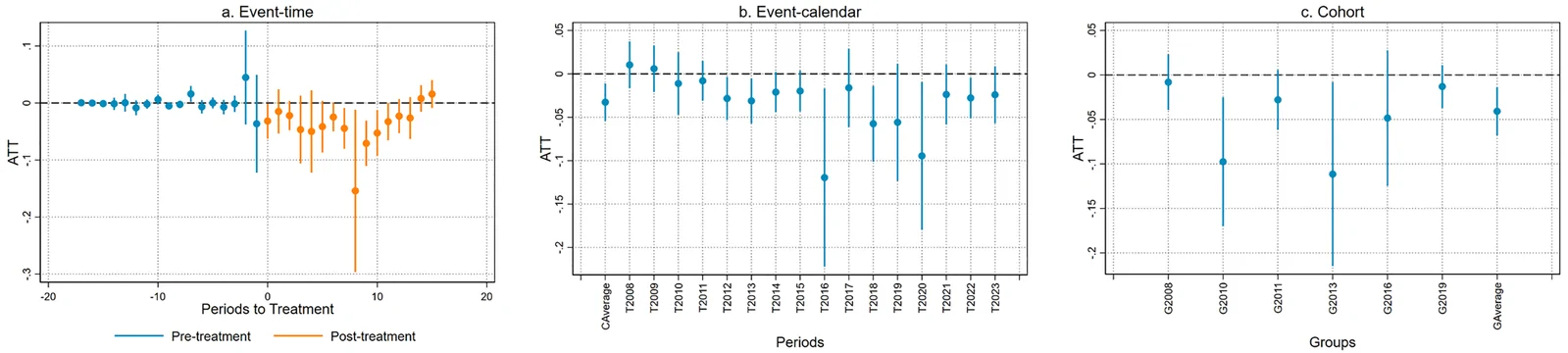
Short-term effects of Buffer Zone establishment on deforestation across estimators. Staggered DiD estimates (Callaway and Sant’Anna [25]) for BZs established 2006-2018. (a) Event-time estimates; (b) Calendar-time estimates; (c) Cohort-level estimates. Outcome: annual deforestation (ha/km^2^). Bandwidth ± 5 km. Bars indicate 95% confidence intervals.

Regional Conservation Areas (RCAs) exhibit consistently negative yet statistically non-significant effects across all estimators (Fig 6). The absence of detectable impacts aligns with the exploratory nature of this analysis. With nine RCAs in the sample and relatively short post-treatment windows in several instances, statistical power is limited. Nevertheless, the direction of the estimates aligns with a potential conservation effect, and their lack of statistical significance should not be interpreted as evidence of ineffectiveness. RCAs constitute a governance mechanism of increasing relevance in the Peruvian Amazon, operating at subnational levels under regional government authority. Evidence from other Amazonian contexts indicates that subnational and locally governed conservation regimes can match the effectiveness of nationally administered areas when institutional capacity and community engagement are adequately developed [54,55]. Future research employing larger samples and longer post-treatment windows should reassess this category as the RCA network continues to expand.

**Fig 6:**
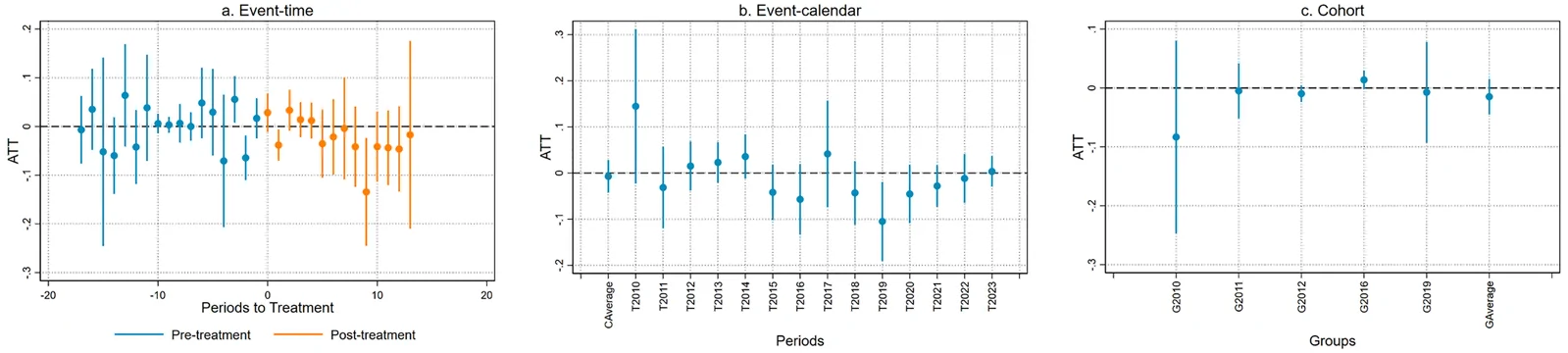
Short-term effects of Regional Conservation Area establishment on deforestation across estimators. Staggered DiD estimates (Callaway and Sant’Anna [25]) for RCAs established 2006-2018 (n = 9; exploratory). (a) Event-time estimates; (b) Calendar-time estimates; (c) Cohort-level estimates. Outcome: annual deforestation (ha/km^2^). Bandwidth ± 5 km. Bars indicate 95% confidence intervals.

Additional estimates by use type indicate negative but non-significant coefficients for both direct-use and indirect-use categories (S1 Fig). Parallel trends hold for direct-use PAs and for BZs of both types. Two additional robustness checks were conducted. First, a bandwidth falsification test estimated the main model using both ±5 km and ±10 km windows around boundaries; the estimated effects remain consistent across both specifications, and the parallel trends assumption continues to hold. Second, a donut-type DiD was applied by excluding observations within ±0.5 km and ±1 km of the boundary, confirming that the results are not sensitive to treatment assignment near the boundary. In all cases, the estimated effects remain stable and non-significant for PAs and RCAs, while BZs show modest reductions consistent with the main findings. Detailed results are reported in S2 Text.

Taken together, the short-term analysis indicates that conservation policies in Peru require time to consolidate, as newly established PAs and RCAs show no immediate measurable effects, while BZs begin to show impacts only after several years. This aligns with our long-term findings showing that conservation performance develops progressively.

## Discussion

Our findings confirm the initial hypothesis: PAs significantly reduce deforestation in the long term, while BZs act as complementary instruments by absorbing external pressures. The absence of statistically significant short-term effects implies that conservation benefits require an implementation lag to consolidate, as ecological recovery and governance stabilization must mature before measurable impacts emerge [43,44]. Protected Areas are not static interventions, and their efficacy in mitigating habitat loss often takes time to materialize [44]. Event-study evidence similarly shows that conservation benefits unfold gradually rather than appearing immediately after establishment [43]. Failing to account for this temporal dimension can produce inconclusive results in effectiveness studies, since meaningful conservation outcomes often require institutional changes that extend well beyond the initial designation [56]. The long-term stabilization of deforestation reduction therefore depends on the maturation of both institutional and ecological processes [42].

Between 2001 and 2023, PAs lost only 0.5% of their forest cover, compared to 5.25% in surrounding BZs. This pattern is consistent with Dourojeanni [29], who noted that accumulated deforestation in direct-use PAs in the Peruvian Amazon was six times higher than in strictly protected indirect-use areas, and illustrates the displacement of deforestation pressures toward peripheral zones. This displacement effect has been documented elsewhere: Ford et al. [20] found deforestation leakage in nearly half of 120 pan-tropical and subtropical PAs, while Lui and Coomes [19] showed that deforestation rates rise gradually from the reserve interior toward the outer edge of the BZ across 60 global natural reserves. At the national level, this pattern is consistent with prior evidence that PAs reduce deforestation in Peru relative to unprotected land [26–28]. The role of external economic drivers in sustaining pressure on PA peripheries is further illustrated by Llopis et al. [57] in Madagascar, where the expansion of cash crops such as cloves and vanilla maintained persistent deforestation pressure on surrounding forest despite reduced loss rates within PAs. By extending the temporal scope to 2023, our analysis demonstrates that BZs themselves can mitigate deforestation, thereby playing a more active role than usually acknowledged.

The analysis of heterogeneous effects highlights the case of Tingo María National Park. Despite being the smallest PA in the long-term sample, it shows a significant reduction in deforestation, exceeding 9 ha/km^2^, comparable to that observed in Tambopata National Reserve. This finding aligns with Wolf et al. [58], who argue that smaller PAs may deliver stronger conservation outcomes. Tingo María also illustrates how, even with extractive concessions operating within its BZ, deforestation remains largely contained in that space and has minimal impact on the PA (Fig 7B). By contrast, extensive areas such as Pacaya-Samiria show no significant impacts, likely driven by low external pressures (Fig 7A).

**Fig 7:**
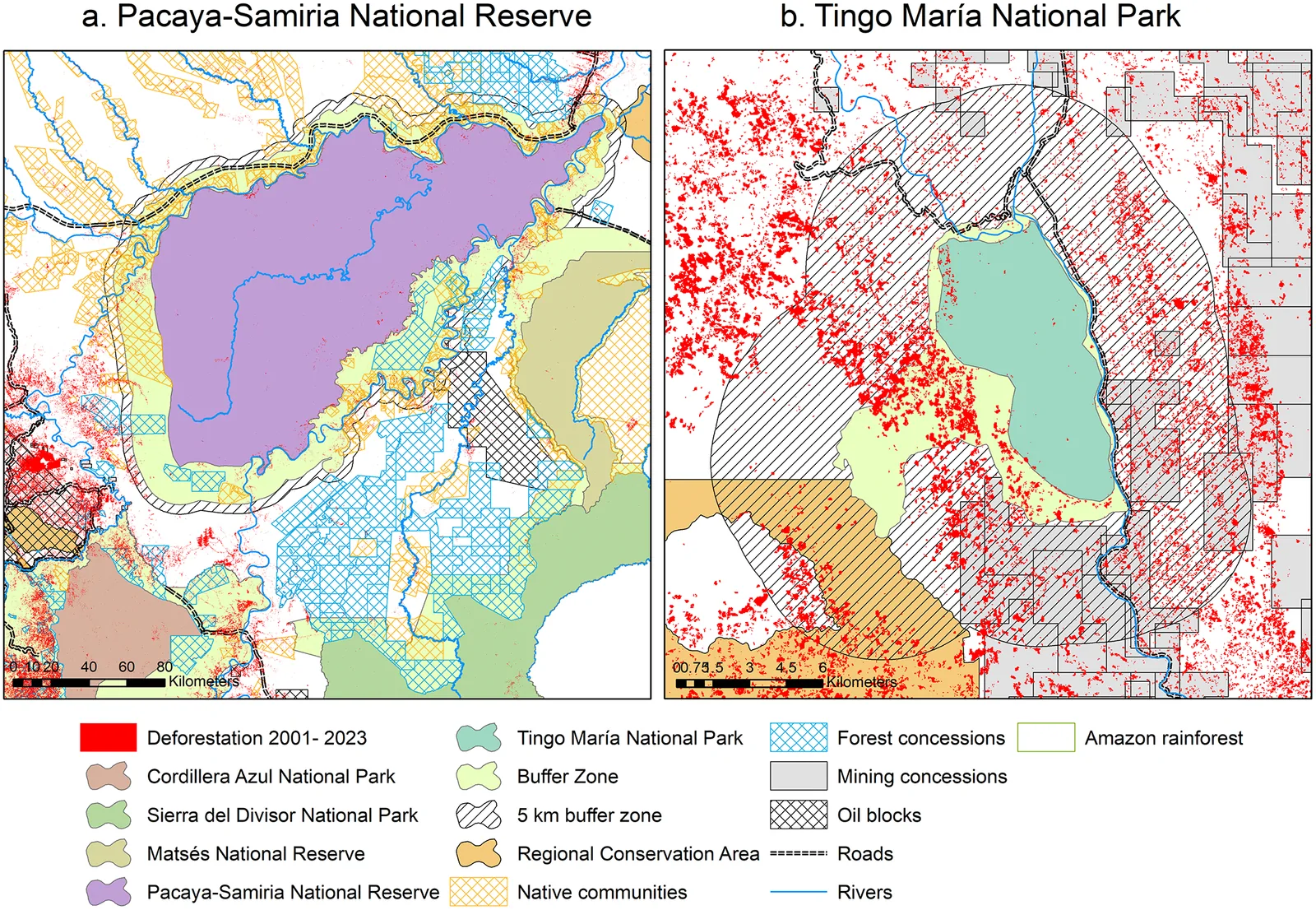
Deforestation patterns (2001-2023) in Pacaya-Samiria National Reserve and Tingo María National Park. Sources: Authors’ elaboration based on geospatial data from INGEMMET, SERNANP, MINAM, Instituto del Bien Común, MTC, Instituto Geográfico Nacional, used with permission (see S1 Table for details).

In the Buffer Zone of Tambopata National Reserve, deforestation pressures concentrate along the Interoceanic Highway and in mining concessions, but remain largely contained within the BZ, supporting evidence that PAs can slow the advance of infrastructure-related deforestation [59–61] (Fig 8).

**Fig 8:**
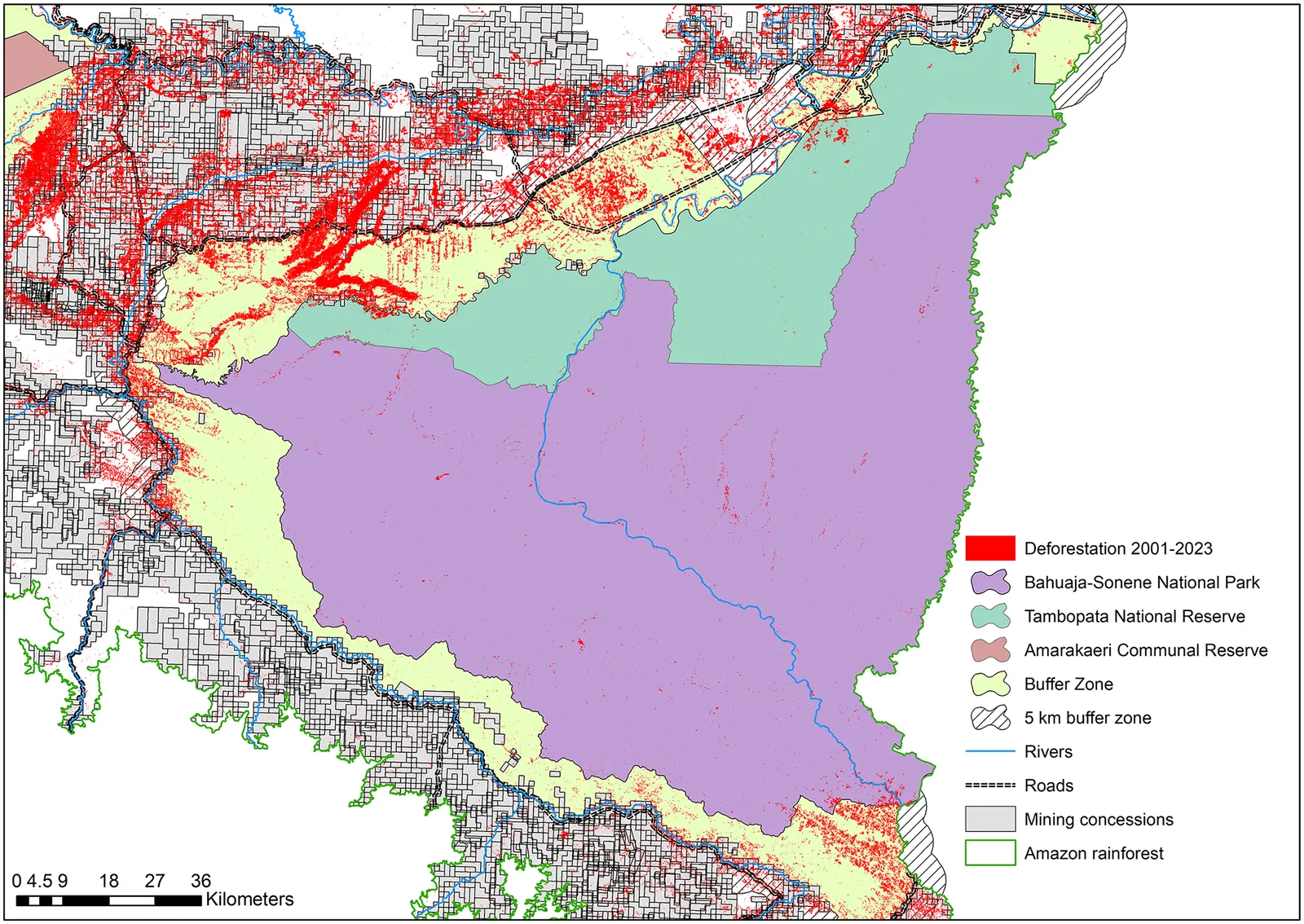
Deforestation patterns (2001-2023) in Tambopata National Reserve and Bahuaja Sonene National Park. Sources: Authors’ elaboration based on geospatial data from INGEMMET, SERNANP, MINAM, Instituto del Bien Común, MTC, Instituto Geográfico Nacional, used with permission (see S1 Table for details).

The Peruvian Society for Environmental Law [62] had already reported illegal mining activity in Tambopata, linked to deforestation and mercury contamination. Similarly, USAID [63] notes that at least three PAs included in this analysis (Bahuaja Sonene National Park, Tingo María National Park, and Santiago Comaina Reserved Zone) are affected by illegal mining activity in their BZs. In line with these findings, the Buffer Zone of Bahuaja Sonene shows much lower levels of external pressure, which may explain the absence of statistically significant effects. Nevertheless, the BZ appears to perform its containment function, as deforestation does not extend into the PA (Fig 8).

The role of governance and community-based management is also evident in the Yanesha Communal Reserve, which shows consistent reductions in deforestation both inside the PA and in its BZ. This contrasts with Orihuela and Pérez [28], who applied geographic RDD across the Peruvian Amazon and found that Yanesha was effective within its boundaries but showed no significant impact in its BZ, largely because much of that peripheral area had already been deforested before their study period. The results presented here highlight that BZs can play a more active role in mitigating deforestation than previously documented for this specific case.

The effectiveness of participatory management is further supported by Schleicher et al. [64], whose propensity score matching analysis across the Peruvian Amazon found that Indigenous territories and Conservation Concessions outperformed state-administered PAs on average, recording lower rates of deforestation and forest degradation. This is consistent with the global meta-analysis by Porter-Bolland [65], who found that community-managed forests exhibit lower and less variable annual deforestation rates than strictly protected PAs.

Boundary-level governance interactions also shape conservation outcomes elsewhere. Amin et al. [66] found in the Brazilian Amazon that a 10% increase in the surface area of indigenous lands was associated with approximately 10.08 km^2^ of avoided deforestation in surrounding areas, indicating positive spillover effects. As Schleicher et al. [64] argue, differentiating between these governance arrangements as a homogeneous baseline can obscure the actual effect of specific land-use regulations, such as logging or mining concessions, on forest integrity [65–67].

A comparison with Rojas-Briceño et al. [47] illustrates how methodological choices matter. While their index of cumulative deforestation rates classified Yanesha, San Matías-San Carlos Protection Forest, and Alto Mayo Protection Forest as moderately effective or ineffective, the RDD approach applied here detects significant local containment effects at boundaries. This divergence may be explained by differences in methodological approach: while Rojas-Briceño et al. [47] analyze aggregate deforestation rates using each PA as the unit of observation, our framework estimates local causal effects at legal boundaries, incorporating geographic controls and using 1 km^2^ grid cells as the unit of analysis.

Regarding welfare, households in BZs report lower income and expenditure levels, most pronouncedly near indirect-use PAs where restrictions are stricter. Although coefficients are modest, they are statistically significant and consistent with Orihuela and Pérez [28]. Several mechanisms may explain this pattern. First, BZ regulations, defined in each PA’s Master Plan, restrict or prohibit activities such as logging, agriculture expansion, and resource extraction that would otherwise constitute income sources for local households [4,29]. These restrictions are more stringent near indirect-use PAs, which likely explains the stronger negative welfare effects observed in those areas (Table 6). Second, BZs in the Peruvian Amazon are often located in geographically remote areas with limited access to markets, public services, and infrastructure, which constrains households’ ability to substitute restricted activities with alternative income sources [35,36]. Third, while direct-use PAs allow controlled activities compatible with conservation, the income generated by these activities tends to be unevenly distributed and may not reach the majority of BZ households [37,38]. These mechanisms suggest that the observed welfare costs are not a direct consequence of conservation per se, but rather of the absence of compensatory policies that provide viable economic alternatives to affected households [33,40,41]. A more comprehensive welfare assessment would require additional indicators, such as access to water, health, and education, since income and expenditure capture only part of household living conditions.

The equity considerations are especially pronounced in proximity to indirect-use PAs, where restrictions on resource access are most severe and households face the sharpest trade-offs between conservation adherence and economic activity. Addressing these costs requires policy responses that extend beyond generic livelihood support and target the specific mechanisms through which welfare is affected in high-restriction settings.

Short-term results provide further insights into the temporal dynamics of conservation. The staggered DiD analysis shows that neither PAs nor RCAs established after 2001 have immediate effects, while certain BZ cohorts exhibit modest but significant reductions eight to ten years after establishment. This pattern reinforces evidence that conservation outcomes accumulate gradually [43] and depend on broader governance factors such as institutional coordination and political stability. A similar lag has been documented by Desbureaux et al. [15], who found that the effect of management partnerships in Africa was limited during the first five years but increased steadily between years six and ten. Li et al. [42] similarly argue that measurable effects typically emerge at least a decade after establishment, once administrative capacity and ecosystem recovery consolidate.

Spatial analysis also reveals interactions with extractive activities: in BZs near mining or oil concessions, deforestation pressures are evident, and leakage effects toward neighboring communities cannot be ruled out (S2 Fig). He et al. [67] found that mining-induced forest loss within a 10 km buffer around PAs, in North America and Asia, was 2.5 times higher than within the PAs themselves, underscoring how existing regulations often fail to contain extractive pressures at the periphery. The trade-offs between conservation and local socioeconomic development are shaped by spatial dynamics that extend beyond administrative boundaries, reinforcing the need for multidimensional welfare indicators such as education and sanitation [68,69].

Finally, the results also show that the permitted use type within PAs, whether direct or indirect, influences their effectiveness but does not determine it alone [70]. Although direct-use PAs and their associated BZs tend to show greater reductions in deforestation than their indirect-use counterparts, the differences in magnitude are modest. This contrasts with Dourojeanni [70], who found that accumulated deforestation in direct-use PAs in the Peruvian Amazon was six to ten times higher than in strictly protected indirect-use areas, illustrating that the relative effectiveness of each use type can vary substantially across studies and contexts.

Local governance, extractive industry, and institutional capacity help explain this variability [41,71], and in politically unstable settings, such pressures are often exacerbated [72]. Shah et al. [71] found that PA networks are most effective in countries with stronger governance quality, while Wuepper et al. [23] identified enforcement and regulation stringency as the strongest predictors of conservation success across a global sample. In the Peruvian context, Rasmussen [41] attributes successful cases to a process of negotiated coexistence, in which PA managers move away from top-down models toward a reciprocal arrangement between state authorities and local organizations. Such arrangements can prove fragile under external shocks, as Neugarten et al. [72] showed for Madagascar, where community-managed forests suffered disproportionately more than state-run forests after a political crisis disrupted centralized support.

### Study limitations

Some limitations of this study should be acknowledged. First, regarding data constraints, the welfare analysis relies on the ENAHO, a nationally representative household survey that is not specifically designed to capture socioeconomic particularities of populations living within or adjacent to PAs. Its sampling density within Protected Areas is limited, which is why the welfare analysis is restricted to BZs and cannot be extended to PAs’ interiors. Furthermore, ENAHO does not capture income from activities that are highly relevant in conservation contexts, such as ecotourism, carbon credit schemes, or REDD+ and MERESE mechanisms, which may lead to an underestimation of welfare benefits associated with compatible land uses. Second, following Orihuela and Perez [28], a cell is classified as treated if the PA or BZ boundary intersects it. As a result, cells straddling the boundary contain territory on both sides, which may introduce some degree of treatment misclassification near the boundary. The donut-type RDD robustness checks confirm that this classification decision does not affect the main results (see S1 Text). Cases where two PAs share a boundary are handled by dissolving the shared limit and assigning each cell to the nearest area [29,47]. Third, the temporal structure of the data creates an unavoidable gap. Areas established between approximately 2001 and 2005 could not be included in either analysis, as they lack sufficient pre-treatment data for the DiD and are too recent for the cross-sectional RDD. This limits the representativeness of the sample with respect to the full population of PAs in the Peruvian Amazon. Fourth, the econometric models rely on identifying assumptions, namely continuity of covariates at the boundary for the RDD, and parallel trends for the DiD. Although these assumptions are empirically tested and supported (see robustness checks), they may not fully capture the heterogeneity of conditions across individual PAs. Fifth, deforestation control is only one dimension of PA effectiveness. PAs may also deliver ecosystem services, biodiversity protection, and cultural values that are not captured by forest cover metrics [73,74]. In some cases, low deforestation rates may reflect geographic inaccessibility [10], while illegal extractive activities can cause severe ecological damage without producing detectable changes in forest cover [75]. Sixth, institutional constraints, including weaknesses in policy implementation, insufficient monitoring of PAs’ Master Plans, and tensions arising from decentralized governance, independently limit conservation outcomes and fall outside the scope of what causal inference methods can estimate [41,76]. Seventh, the exploratory examination of RCAs reflects a genuine data limitation. Given a sample comprising 9 RCAs and post-treatment periods that are frequently too brief to capture emerging conservation effects, the statistical power of this component of the analysis is insufficient to draw strong conclusions. Although the estimates consistently trend negative, implying a potential conservation effect, subsequent evaluations employing larger samples and extended time horizons are required to assess RCA performance with greater confidence.

### Policy implications

The results reveal patterns that carry distinct implications for each governance category, making a universal policy approach unlikely to be effective given the heterogeneity of conservation arrangements in the Peruvian Amazon.

For nationally administered PAs, the long-term analysis confirms that legal protection significantly reduces deforestation and that effectiveness varies substantially by category. Communal Reserves reduce deforestation by up to 7.5 ha/km^2^ and their associated BZs by up to 9.7 ha/km^2^, the strongest result in the entire sample. Protection Forests achieve reductions of up to 4.1 ha/km^2^ in the PA and containment in the adjacent BZs such as Alto Mayo. In contrast, National Sanctuaries and Reserved Zones show no significant effects. Smaller, higher-pressure sites, such as Tingo María National Park, with reductions of 5.7 to 9 ha/km^2^, outperform large remote areas such as Pacaya Samiria and Manu National Parks, where effects are absent, indicative of limited external pressure rather than institutional failure. These contrasts suggest that conservation policy should prioritize management investment and enforcement in areas where external pressures are demonstrably high. Sustaining SERNANP’s capacity to enforce Master Plans and monitor compliance in high-pressure zones is therefore a more targeted priority than broad institutional strengthening [47,77]. The strong performance of Communal Reserves also points to the value of co-management arrangements with indigenous organizations, though Barletti y Rolando [78] caution that these regimes can generate unequal transfers of responsibilities to indigenous communities, which requires active governance design to address.

For Buffer Zones, the results present a dual challenge. BZs reduce deforestation by 1.1 to 1.5 ha/km^2^ in the long term. In the short term, although the effects are not statistically significant, BZs exhibit a more pronounced pattern than PAs and RCAs, whose coefficients remain near zero. The reductions for BZs peak at years 8 and 9 following establishment, with the largest cumulative magnitude (0.111 ha/km^2^) recorded for BZs of PAs creates in 2012. However, households within BZs adjacent to indirect-use PAs experience income losses of up to 1.2 times the poverty line and expenditure reductions of up to 0.6 times the poverty line, considerably higher than the 0.4 and 0.3 times the poverty line observed near direct-use areas. This pattern evidence stricter resource restrictions near indirect-use PAs, representing the primary equity concern highlighted here. Policy design for BZs should thus pursue a two-track approach: reinforcing the containment function, primarily in BZs near National Reserves such as Tambopata, where illegal extractive activities persist [30], and implementing targeted compensation for the most affected households. Mechanisms such as Payments for Ecosystem Services (PES), REDD + , and MERESE schemes offer direct channels for this, provided benefit distribution is transparent and reaches affected communities [34]. Morgans et al. [36] show for Indonesia that coupling conservation restrictions with programs supporting alternative livelihoods simultaneously reduces deforestation and improves household welfare, a model directly applicable to the high-restriction BZ context adjacent to indirect-use PAs.

For Regional Conservation Areas, the consistently negative yet non-significant effects across event-time, calendar, and cohort estimators suggest that conservation processes may be underway but have not yet produced detectable outcomes. The policy priority is therefore institutional strengthening at the regional government level: developing Master Plans where absent, enhancing deforestation monitoring capacity, and ensuring adequate financing for management operations. Cano et al. [79] evaluate the Maijuna-Kichwa RCA in Loreto and report improvements in resource abundance and community safety, but also persistent challenges related to government support, communication, and equitable participation that must be resolved before these areas can consolidate their conservation function.

Across all three categories, income and expenditure metrics are insufficient to assess whether conservation and welfare goals are being met simultaneously. The development of multidimensional indicators that capture food security, access to services, and governance within communities would yield a more comprehensive assessment [80]. As noted by Dourojeanni [70], conservation policy must be integrated into broader territorial planning to prevent economic development and extractive activities from undermining ecological integrity. Explicit incorporation of gender equity and intergenerational participation into design criteria across all interventions is warranted, given the differential welfare impacts linked to household composition and livelihood strategies [34,41].

## Conclusion

We evaluated the effects of establishing PAs, BZs, and RCAs on deforestation and welfare in the Peruvian Amazon, distinguishing between short- and long-term outcomes. Over the long term, both PAs and BZs achieve significant reductions in deforestation. Specifically, PAs reduce forest loss by 1.3 to 2.8 ha/km^2^, while BZs reduce it by 1.1 to 1.5 ha/km^2^. These results confirm that both instruments function as effective conservation tools for maintaining forest cover.

The analysis also shows that direct-use categories, such as Protection Forests and Communal Reserves, are notably effective, including within their Buffer Zones. In contrast, large PAs such as Pacaya Samiria and Manu do not present significant reductions, likely because they are located in regions subject to lower extractive pressures compared to smaller areas such as Tingo María National Park.

On the welfare side, households residing within BZs experience lower per capita income and expenditure levels. This decline appears driven by restricted access to resources and services, especially in zones adjacent to indirect-use PAs, where restrictions are more stringent.

In the short term, no significant effects are identified, except in certain BZs, which begin to show modest but measurable reductions over time. This short-term dynamics imply that conservation policies require a consolidation period before producing detectable impacts.

From a methodological perspective, this study introduces the staggered Difference-in-Differences model for the first time in the Peruvian context and provides exploratory evidence on the role of RCAs. Nonetheless, results should be interpreted with caution due to limitations, such as the exclusion of areas created near the temporal threshold between both analyses and the technical challenges of working with high-resolution geospatial data.

These findings reinforce the importance of moving beyond legal designation toward adaptive and participatory management models. Strengthening local governance and community engagement is essential to ensure that conservation outcomes are sustained over time. Future efforts should also incorporate multidimensional indicators that allow for a more holistic evaluation of the ecological and social performance of Protected Areas.

## Supporting information

S1 Table Data sources and study variables. (DOCX)

S2 Table Heterogeneous effects: Deforestation impact by PA. (DOCX)

S3 Table Heterogeneous effects: Deforestation impact by PA associated with each Buffer Zone. (DOCX)

S1 Fig Short-term impact on deforestation by PA use type and associated BZ use type. (TIF)

S2 Fig Annual deforestation points (2001–2023) in selected Protected Areas established after 2001. Areas shown include (a) Matsés National Reserve, (b) Chayu Naín Communal Reserve and Cordillera de Colán National Sanctuary, and (c) Pampa Hermosa National Sanctuary. Sources: Author’s elaboration based on geospatial data from MINAM, SERNANP, Instituto del Bien Común, SERFOR, INGEMMET, Perú Petro, MTC, and Instituto Geográfico Nacional, used with permission.
(TIF)

S1 Text Robustness tests for the regression discontinuity design. (DOCX)

S2 Text Robustness tests for the staggered difference-in-differences model. (DOCX)
