# Supplementary material for: Causal inference in conservation policy: Evaluating the effects of Protected Areas on deforestation and well-being in the Peruvian Amazon
Source: PLoS One. 2026 Oct 14;21(10):e0337468. doi: 10.1371/journal.pone.0337468 (PMC13667790; doi:10.1371/journal.pone.0337468)
Supplement: Supplementary file 1 [file pone.0337468.s009.pdf]

Reviewer Observations Table: PONE-D-25-59578

Causal inference in conservation policy: Evaluating the effects of Protected Areas on deforestation and well-being in the Peruvian Amazon

N°	Observation	Authors' Response
**JOURNAL REQUIREMENTS**		
**JR-1**	Ensure the manuscript meets PLOS ONE's style requirements, including file naming conventions.	The manuscript has been reformatted to comply with PLOS ONE's style requirements.
**JR-2**	Decide on a data sharing plan before acceptance. All data must be freely accessible upon publication (except where restricted by an ethics board). If open data policy cannot be met, revise the Data Availability Statement with justification and request an exemption.	We have completed the data sharing plan. All processed data and analysis scripts that underlie the study's findings are publicly accessible on Zenodo at https://doi.org/10.5281/zenodo.22244470, including all processed datasets required to reproduce the models. Raw source data are publicly available via their respective institutional portals, as described in S1 Table; raw ENAHO microdata are not redistributed directly but can be downloaded on demand from INEI's official microdata portal using the provided download script. The Data Availability Statement has been updated to reflect these provisions.
**JR-3**	Amend the authorship list in the manuscript file to include: Teresa Liliana Paredes Quiroz and Karen Ilse Eckhardt Rovalino.	The authorship list in the manuscript file has been amended to include the full legal names.
**JR-4**	Amend the manuscript submission data (via Edit Submission) to include: Teresa L. Paredes and Karen I. Eckhardt.	The manuscript submission metadata has been updated to include Teresa L. Paredes and Karen I. Eckhardt.
**JR-5**	Figure 1 contains copyrighted images. PLOS ONE requires all content to be published under CC BY 4.0. Either: (a) obtain written permission from the copyright holder to publish under CC BY 4.0 using the Content Permission Form, or (b) remove or replace the figure.	Figure 1 is an original schematic diagram created entirely by the authors to illustrate the treatment/control zone assignment used in the RDD design, as indicated in the figure caption ("Authors elaboration"). It does not reproduce or derive from any copyrighted, proprietary, or third-party map, image, or dataset. Therefore, no permission request or Content Permission Form applies to this figure.
**JR-6**	Figures 2, 7, 8, and S2 contain map/satellite images that may be copyrighted (e.g., Google Maps, Street View, Earth). Either: (a) obtain permission from the copyright holder using the Content Permission Form, or (b) remove or replace the figures with public domain sources.	We have obtained written permission from relevant copyright holders (MINAM, SERNANP, Instituto del Bien Común, SERFOR, INGEMMET, PerúPetro, MTC and IGN) to publish Figures 2, 7, 8, and S2 Fig under CC BY 4.0 license. The corresponding permission forms and official written authorizations (in Spanish and English) are included as supporting files with this submission. The figure captions have been updated to credit each institutional source accordingly.
**JR-7**	If reviewers recommend citing specific previously published works, review and evaluate those publications to determine whether they are relevant and should be cited. There is no requirement to cite them unless the editor indicates otherwise.	The reviewers did not recommend specific published works to cite. We have nonetheless updated and expanded the literature discussed throughout the manuscript, particularly with additional recent global references, as detailed in our responses to Reviewer #2 and Reviewer #3 below.

REVIEWER #1		
**R1-1**	The identification strategy requires clearer justification, particularly regarding the validity of the RDD assumptions near PA and BZ boundaries.	We have strengthened the justification of the RDD identification strategy in three locations of the manuscript:<br>(a) In the Empirical Strategy section, we expanded the paragraph describing the RDD assumption (lines 263- 277) to explain why it is plausible in this specific context: PA and BZ boundaries in Peru are defined through administrative and legal instruments (Law N° 26834) rather than pre-existing socioeconomic gradients; the model includes comprehensive geographic and accessibility controls; boundary fixed effects capture area-specific unobservables; and the design follows previous geographic RDD applications in Colombia and Peru where the same assumption has been argued to hold<br>(b) Following the description of the running variable (lines 251-258), we added a paragraph clarifying that the use of distance to the boundary as the running variable is appropriate because PA and BZ boundaries were not drawn based on proximity to populated areas or pre-existing land-use intensity, making the cutoff plausibly exogenous to the outcomes of interest.<br>(c) In the Results section, we expanded the robustness checks paragraph (lines 473-481) to explicitly connect the donut RDD, placebo tests, and artificial boundary tests to the validation of the core RDD assumption, noting that the absence of effects at false boundaries confirms that covariates do not change discontinuously at the true legal boundary
**R1-2**	The distinction between short-term and long-term effects is interesting, but the temporal thresholds used to define these periods are not sufficiently explained.	We have clarified the temporal thresholds underlying the long-term/short-term distinction in three locations of the manuscript:<br>(a) In the Introduction (lines 142-152), we added a brief explanation indicating that the 2001 threshold is driven by data availability, specifically, the onset of high-resolution annual deforestation records, and directing the reader to the Empirical Strategy section for the full technical justification<br>(b) At the end of the Empirical Strategy section (lines 337-351), we replaced the brief closing remark with a detailed explanation of: why areas established before 2001 cannot be evaluated with a panel design; the substantive meaning of *long-term* (minimum 22 years of accumulated protection by 2023); why the DiD sample is restricted to 2006-2018 (minimum five pre- and post-treatment years); and the clarification that the temporal labels do not imply a methodological hierarchy between the RDD and DiD<br>(c) In the Short-term DiD subsection (lines 487-492), we expanded the sentence describing the sample window to explicitly justify both bounds: the lower bound of 2006 (minimum pre-treatment window to test parallel trends) and the upper bound of 2018 (minimum five post-treatment years for effects to materialize)
**R1-3**	Welfare impacts are reported, yet the mechanisms through which PAs and BZs affect household income and expenditure remain underdeveloped.	We have expanded the welfare discussion in the Discussion section (lines 651-666) to articulate the mechanisms through which PAs and BZs affect household income and expenditure. Specifically, we identify three plausible channels: (a) regulatory restrictions on resource extraction and agricultural expansion established in PA Master Plans, which are more stringent near indirect-use PAs and directly limit household income sources; (b) geographic remoteness and limited market access in BZ areas, which constrains the ability of

		households to substitute restricted activities; and (c) the uneven distribution of income from compatible activities such as ecotourism, which does not systematically benefit BZ households. We also clarify that the observed welfare costs likely reflect the absence of compensatory policies rather than conservation itself and retain the caveat that income and expenditure are partial welfare measures
**R1-4**	The negative welfare effects near indirect-use PAs raise important equity concerns, but the analysis stops short of exploring policy-relevant mitigation strategies.	We have addressed this comment in two ways. First, the welfare paragraph in the Discussion (lines 667-671) was extended to flag explicitly the equity concerns specific to indirect-use PAs. Second, a dedicated "Policy Implications" sections was created after Study Limitations, consolidating and reorganizing policy-relevant content that was previously dispersed across the Discussion and embedded within Study Limitations. The new section identifies three targeted areas of intervention: compensation mechanisms including PES, REDD+, and MERESE schemes, supported by [36] Morgans et al (2024), who showed for Indonesia that combining conservation restrictions with livelihood support simultaneously reduces deforestation and improves household welfare, conservation agreements and co-management frameworks adapted to local governance structures; and strengthening SINANPE's operational capacity alongside multidimensional welfare indicators. Gender equity is explicitly identified as a design criterion across all three.
**R1-5**	The treatment of Regional Conservation Areas (RCAs) is exploratory and underpowered, limiting the strength of conclusions drawn for this category.	We acknowledge this limitation and have addressed it in three locations. First, in the Empirical Strategy section (lines 318–323), we state explicitly that the RCA analysis is exploratory due to the small sample size of 9 RCAs and the accompanying limitations in statistical power. Second, in the Results section (lines 524-536), we broaden the RCA discussion to indicate that non-significance arises from data limitations rather than ineffectiveness and note that the estimated directions align with a conservation effect. We situate the RCA category within the broader literature on subnational conservation governance, drawing on [54] Qin et al. (2024) and [55] Virtanen et al. (2025). Third, in the Study Limitations subsection (lines 742-748), we include a dedicated point on the limited statistical power of the RCA analysis and advocate for future research employing larger samples and longer post-treatment windows
**R1-6**	The study's conceptual novelty is moderate, as similar PA impact evaluations exist in other tropical regions.	We acknowledge the existence of impact evaluations of PAs in tropical regions and thus recognize that the study's conceptual contribution is contextual rather than foundational. The final paragraph of the Introduction (lines 157-165) has been revised to explicitly acknowledge this while detailing aspects unique to the Peruvian context: (i) the inaugural application of the staggered DiD framework to PA evaluation in Peru; (ii) the joint treatment of three governance categories within a single causal-inference framework; (iii) the longest temporal window present in the national literature; and (iv) the simultaneous estimation of ecological and socioeconomic outcomes under a common identification strategy. Rather than asserting broad conceptual originality, the study repositions its contribution at the nexus of methodological rigor and contextual specificity

**R1-7**	The policy implications would benefit from clearer differentiation between national PAs, BZs, and RCAs in terms of governance and management design.	The Policy Implications section has been reorganized and rewritten to derive differentiated recommendations directly from the heterogeneous outcomes observed for each governance category. For nationally administered PAs, recommendations are anchored in results by PA type: the substantial performance of Communal Reserves and Protection Forests under active pressure (up to 9.0 and 6.8 ha/km^2, respectively) contrasts with the lack of effects in large remote areas such as Pacaya Samiria and Manu, indicating that investments in management should be prioritized where external pressures are demonstrably high. For BZs, policy guidance addresses the dual challenge identified in the results: effective long-term deforestation containment alongside concentrated welfare costs near indirect-use PAs, where income losses reach as much as 1.2 times the poverty line. For RCAs, recommendations emphasize the institutional preconditions necessary for effectiveness, drawing on [79] Cano et al. (2026) regarding the Maijuna-Kichwa RCA. A concluding paragraph delineates cross-cutting priorities that apply across all three categories.
**REVIEWER #2**		
**R2-1**	The Discussion section requires further elaboration. A dedicated subsection on study limitations should be included, particularly highlighting data constraints and other factors that may have influenced the analysis.	We have reorganized and expanded the content limitations in the Discussion section by creating a dedicated subsection titled "Study limitations" (lines 711-748), which replaces the two previously dispersed limitation paragraphs. The subsection systematically addresses seven categories of limitations: (a) data constraints related to ENAHO's sampling design and its inability to capture conservation-compatible income sources; (b) potential treatment misclassification arising from the use of 1 km^2 grid cells with partial boundary overlaps; (c) the temporal gap affecting areas established between 2001 and 2005, which could not be included in either analysis; (d) the assumptions underlying both causal inference methods and their potential limitations in highly heterogeneous PA contexts; (e) the restriction of deforestation as the sole effectiveness metric, noting that inaccessibility and illegal extraction may confound results; (f) institutional constraints that independently affect conservation outcomes beyond what the models can estimate; and (g) the exploratory nature of the RCA analysis, as detailed in our response to Comment R1-5.
**R2-2**	The Discussion currently relies heavily on local and regional literature. The authors are encouraged to incorporate more recent and relevant global literature on the topic.	We have incorporated additional recent and global literature throughout the manuscript, including: [8] Visconti et al. (2019), [9] Geldmann (2026), [54] Qin et al. (2024), [55] Virtanen et al. (2025), [56] Ramiandrisoa et al. (2026), [67] He et al. (2025), [68] den Braber et al. (2024), [69] Gaglia Alves et al. (2025), [78] Sarmiento Barletti and Rolando (2024).
**R2-3**	Line 19: Revise to "Study area" only; there is no need to mention "units of analysis" at this point.	The heading of the corresponding subsection in Materials and Methods (line 170) has been revised from "Study area and units of analysis" to "Study area", as recommended. The description of the units of analysis remains in the body of the section where it is introduced in context

**R2-4**	Lines 236-238: This content pertains to methods/analysis and should be relocated to the appropriate section rather than the Results section.	The description of the local conditional means test has been relocated from the Results section to the Long-term analysis, RDD subsection within Empirical Strategy, where it now appears as a methodological step preceding the model specification (lines 278-280).
**R2-5**	Expand the titles of all figures and tables throughout the manuscript. The current titles are too brief and do not provide sufficient information to fully understand the content presented.	The titles of all figures and tables throughout the manuscript have been revised to be more informative. In addition, detailed captions have been added to all figures and tables.
**REVIEWER #3**		
**R3-1**	The introduction could be strengthened by briefly referencing how buffer zones and RCAs compare to other protected area types in the existing literature, even if only conceptually.	We have strengthened the Introduction by incorporating a paragraph (lines 92-98) that briefly positions BZs and RCAs within the broader categories examined in the extant global literature. Conceptually, BZs correspond to the multi-use or transition zones described around strictly protected areas in other regions, where permitted activities are more flexible than in the core area yet constrained by conservation objectives. RCAs are positioned alongside subnational or regionally governed reserves found in decentralized conservation systems, where effectiveness is often linked to local governance capacity and proximity to communities. The comparison remains at a conceptual level, in accordance with the reviewer's suggestion, and does not conflate the specific legal and institutional characteristics of Peruvian categories with analogous arrangements elsewhere.
**R3-2**	In several instances, the cited literature is referenced in a collective manner without differentiation. The manuscript would benefit from a more explicit discussion of these studies, including a brief explanation of their key findings and how they directly support the stated claims.	We have revised the relevant passages throughout the manuscript to differentiate previously block-cited references, replacing collective citations with individual discussions of each study's specific findings and how they directly support the corresponding claims. This includes the following sections: lines 54-91; lines 557-569; lines 571-585; lines 614-635; lines 696-676;
**R3-3**	The assumption of the RDD model, that unobserved variables do not change discontinuously at the boundary, needs stronger justification.	This concern is addressed by the revisions made in response to Reviewer 1, comment 1 (R1-1)
**R3-4**	Justify the choice of a 1 km² grid resolution and discuss potential treatment misclassification arising from partial overlaps with PA or BZ boundaries.	We have addressed this comment in two locations. First, in the Study area subsection (lines 174-180), we justified the choice of 1 km² grid resolution, the source data from MINAM are available at 30 m² but processing the full Amazonian coverage at that granularity would be computationally prohibitive and clarified the treatment assignment rule following [28] Orihuela and Pérez (2019). A cell is classified as treated if the PA or BZ boundary intersects it, with distance assigned as negative for treated and positive for control cells. Second, in the Study limitations subsection (lines 719-725), we discuss the potential for treatment misclassification arising from cells that straddle the boundary, noting that the donut-type RDD robustness checks (S1 Text, Tables 1-2) confirm this does not affect the main results.
**R3-5**	The claim "leaving an empirical gap that this study addresses by extending the temporal horizon and territorial scope of the analysis"	We expanded the empirical gap claim (lines 113-123) with three sentences explaining why it persisted prior to this study: (a) evaluating recently established PAs requires sufficient pre-

	is a strong claim. Add approximately 3-4 sentences explaining the reasons for this gap (data limitations, methodological challenges, etc.).	and post-treatment data, a condition only met as the deforestation record extended further beyond their establishment dates; (b) prior Peruvian research relied on matching methods, which cannot exploit temporal variation or estimate dynamic treatment effects for areas with staggered establishment dates; and (c) Regional Conservation Areas, governed outside the SINANPE framework, have been systematically excluded from the empirical literature despite their growing role in Amazonian conservation governance
**R3-6**	The description of control variables in the text is concise, but without a sufficiently detailed Table S1 and S2, the variables are not easily interpretable.	In S1 Table, we added a footnote specifying the exact set of controls used in each model. In S2 and S3 Tables, we added a footnote identifying the conservation category of each PA, which is necessary to interpret the heterogeneous effects in conjunction with Tables 2-4 of the main manuscript
**R3-7**	There is inconsistency in the citation format throughout the manuscript (e.g., use of square brackets versus parentheses). The reference style should be standardized according to the journal's guidelines.	The citation format has been standardized throughout the manuscript to use square brackets consistently.
**R3-8**	The images are not well-organized; it is recommended to arrange them neatly within the text. Images also need higher resolution.	Figures have been updated to higher resolution and reorganized within the manuscript to appear immediately following their first mention in the text, in accordance with PLOS ONE formatting guidelines.
**R3-9**	Minor language edits are needed, including inconsistent capitalization throughout the manuscript.	We conducted a full language and style review of the manuscript, correcting inconsistent capitalization and other language inconsistencies throughout.
**R3-10**	Figure captions appear embedded within the main text rather than being placed directly below the figures.	Figure and table captions have been reformatted with bold identifiers and are presented clearly separated paragraphs immediately following their first citation in the text.
**R3-11**	Some sentences are long and slightly overloaded, combining different ideas at once. These should be split or simplified.	We have conducted a full style review to shorten and clarify long or overloaded sentences throughout the manuscript.
**R3-12**	Key robustness results should be reported directly in the manuscript or in formal supplementary tables (e.g., S1-S2), rather than through external Word documents. Additionally, the claim that the estimates satisfy the parallel trends assumption should be supported by more explanation.	We have addressed both points. First, the key robustness results for both the RDD and DiD analyses are now reported directly in the manuscript, with tables and figures retained in the formal supplementary materials (S1 Text and S2 Text). For the RDD, the three robustness checks, donut-type RDD, placebo bandwidth tests, and boundary falsification, are now described with their main findings in the Results section (lines 473-481). For the DiD, the bandwidth falsification test and donut-type DiD are similarly described with results in the text (lines 544-552). Second, we expanded the description of the parallel trends test (lines 499-503) to explain that pre-treatment coefficients were jointly tested for each cohort, that no statistically significant violations were found across PAs, BZs, or RCAs, and that event-time plots further confirm the absence of systematic pre-trends.
**R3-13**	Regression models should be numbered for clarity and ease of reference.	Both regression specifications in the Empirical Strategy section have been numbered to improve clarity. The RDD specification is designated as Equation 1, while the staggered DiD specification is designated as Equation 2.

**R3-14**	The term "Donut-hole radio" should be corrected to "Donut-hole radius".	The term “Donut-hole-radio” has been corrected to “Donut-hole-radius” throughout both the manuscript and the supplementary materials.
