# Supplementary material for: Causal inference in conservation policy: Evaluating the effects of Protected Areas on deforestation and well-being in the Peruvian Amazon
Source: PLoS One. 2026 Oct 14;21(10):e0337468. doi: 10.1371/journal.pone.0337468 (PMC13667790; doi:10.1371/journal.pone.0337468)
Supplement: Supplementary file 2 [file pone.0337468.s001.docx]

**S1 Table.** Data Sources and Study Variables (2001- 2023)

| **Analysis** | **Variable** | **Description** | **Unit of measurement** | **Source** |
| --- | --- | --- | --- | --- |
| **Deforestation** | Grid location | Centroid coordinates of 1 km² grid cells across the Peruvian Amazon forest | Latitude/Longitude | Own elaboration based on forest/non-forest map - MINAM |
|  | Deforestation | Area deforested per grid cell | Hectares per km² | MINAM |
|  | Altitude | Average altitude per grid cell | Meters above sea level | USGS Earth Explorer |
|  | Slope | Average slope per grid cell | Degrees | USGS Earth Explorer |
|  | Temperature | Average temperature per grid cell | °C | WorldClim |
|  | Precipitation | Average precipitation per grid cell | Millimeters | WorldClim |
|  | Soil quality | Average soil quality per grid cell | Scale [1-7] | FAO |
| **Well-being** | Household location | Centroid coordinates of the populated center where the household is located | Latitude/Longitude | ENAHO Module 1 - INEI |
|  | Income/Expenditure | Real monthly per capita income or expenditure relative to the poverty line (Orihuela & Pérez, 2019) | Ratio to poverty line | ENAHO Module 34 - INEI |
|  | Gender | Gender of the household head | Dummy: 1 = Male, 0 = Female | ENAHO Module 3 - INEI |
|  | Education level | Highest level of education attained by the household head | Scale [0-12] | ENAHO Module 3 - INEI |
|  | Household size | Total number of household members | Count [1-17] | ENAHO Module 34 - INEI |
| **Common to both dimensions** | Treatment condition | Classification of observation by location: inside PA, buffer zone, or regional conservation area | Dummy: 1 = Inside, 0 = Outside | Own calculation based on SERNANP - GeoANP and INEI |
|  | Distance to PA/Buffer/ACR boundary | Euclidean distance to the protected area boundary (negative = inside, positive = outside) | Kilometers | Own calculation based on SERNANP- GeoANP and INEI |
|  | Amazon forest area | Territorial baseline for spatial processing |  | Own elaboration based on forest/non-forest map - MINAM |
|  | Distance to roads | Distance to nearest road | Kilometers | MTC |
|  | Distance to rivers | Distance to nearest river | Kilometers | Instituto Geográfico Nacional |
|  | Distance to mining concessions | Distance to nearest mining concession | Kilometers | GEOCATMIN - INGEMMET |
|  | Distance to forestry concessions | Distance to nearest forestry concession | Kilometers | SERFOR |
|  | Distance to oil blocks | Distance to nearest oil block | Kilometers | Perupetro |
|  | Distance to native communities | Distance to nearest native community | Kilometers | Instituto del Bien Común |

Note: Variables under *Deforestation* dimension are used in the deforestation analysis only; variables under *Wellbeing* dimension are used in the welfare analysis only; variables under *Common to both dimensions* are used in both analyses. Control variables for the deforestation models include: altitude, slope, temperature, precipitation, soil quality, distance to roads, rivers, mining concessions, forestry concessions, oil blocks, and native communities. Control variables for the welfare models include: gender, education level, and household size.

**Sources:**

FAO. Harmonized World Soil Database v 1.2. https://www.fao.org/soils-portal/data-hub/soil-maps-and-databases/harmonized-world-soil-database-v12/en/

Instituto Geológico, Minero y Metalúrgico [INGEMMET]. Concesiones mineras. https://geocatmin.ingemmet.gob.pe/geocatmin/

Servicio Nacional de Áreas Naturales Protegidas por el Estado [SERNANP]. GeoANP: Visor de las Áreas Naturales Protegidas. https://geo.sernanp.gob.pe/visorsernanp/.

Instituto del Bien Común [IBC]. Comunidades nativas. https://ibcperu.org/mapas

Instituto Geográfico Nacional [IGN]. Ríos y quebradas. https://www.geoidep.gob.pe/instituto-geografico-nacional

Instituto Nacional de Estadística e Informática [INEI]. Encuesta Nacional de Hogares (ENAHO). https://proyectos.inei.gob.pe/microdatos/

Ministerio de Transportes y Comunicaciones [MTC]. Red vial. https://portal.mtc.gob.pe/transportes/caminos/normas_carreteras/informacion_espacial.html

Ministerio del Ambiente [MINAM]. Pérdida de bosques (2001-2023). https://geobosques.minam.gob.pe/geobosque/view/descargas.php?122345gxxe345w34gg

Perupetro. Lotes petroleros. https://geoportal.perupetro.com.pe/home/

Servicio Nacional Forestal y de Fauna Silvestre [SERFOR]. Concesiones Forestales.https://geo.serfor.gob.pe/visor/

USGS EarthExplorer. Altitude and slope.https://earthexplorer.usgs.gov/

WorldClim. Temperature and precipitation. https://www.worldclim.org/data/worldclim21.html
