# Supplementary material for: Causal inference in conservation policy: Evaluating the effects of Protected Areas on deforestation and well-being in the Peruvian Amazon
Source: PLoS One. 2026 Oct 14;21(10):e0337468. doi: 10.1371/journal.pone.0337468 (PMC13667790; doi:10.1371/journal.pone.0337468)
Supplement: Supplementary file 3 [file pone.0337468.s002.docx]

S2 Table. Heterogeneous Effects: Deforestation Impact by PA

|  | Alto Mayo | Bahuaja- Sonene | de Pui Pui | de Tingo María | del Manu | del Río Abiseo | Machupicchu | Pacaya-Samiria | San Matías-San Carlos | Santiago Comaina | Tabaconas-Namballe | Tambopata | Yanachaga-Chemillén | Yanesha | |
| --- | --- | --- | --- | --- | --- | --- | --- | --- | --- | --- | --- | --- | --- | --- | --- |
| 1 km | | | | | | | | | | | | | | |  |
| PA | 0.988 (0.755) | -2.291** (0.984) | 0.361 (0.264) | -5.702 (2.547) | -0.212 (0.278) | -0.820** (0.391) | -1.717** (0.378) | -0.0734 (0.287) | -4.372** (1.944) | -0.076 (0.326) | -0.335 (0.878) | -4.167** (1.549) | 0.415 (0.867) | -3.835*** (1.202) | |
| *R*^2^ | 0.426 | 0.382 | 0.079 | 0.758 | 0.109 | 0.083 | 0.371 | 0.061 | 0.267 | 0.081 | 0.295 | 0.418 | 0.317 | 0.309 | |
| Observations | 1,120 | 1,093 | 255 | 84 | 1,972 | 802 | 117 | 3,156 | 1,222 | 1,612 | 216 | 763 | 429 | 318 | |
| 5 km | | | | | | | | | | | | | | |  |
| PA | -1.001 (1.190) | -4.527*** (1.604) | 0.753* (0.352) | -9.955** (3.011) | -0.282 (0.282) | -0.854* (0.487) | -1.579*** (0.434) | -0.0215 (0.328) | -4.647** (2.144) | -0.304 (0.323) | -0.853 (0.649) | -7.016*** (2.417) | -1.269 (0.899) | -5.681*** (1.124) | |
| *R*^2^ | 0.366 | 0.426 | 0.130 | 0.693 | 0.109 | 0.064 | 0.281 | 0.070 | 0.301 | 0.121 | 0.328 | 0.392 | 0.280 | 0.411 | |
| Observations | 3,144 | 3,494 | 494 | 113 | 5,638 | 1,935 | 284 | 9,840 | 2,682 | 4,362 | 490 | 2,176 | 1,225 | 812 | |
| 10 km | | | | | | | | | | | | | | |  |
| PA | -2.594** (1.210) | -6.494*** (1.673) | 2.123** (0.969) | -9.933** (2.985) | -0.479* (0.280) | 1.722 (1.336) | -2.028*** (0.611) | -0.406 (0.289) | -4.958** (2.180) | -0.401 (0.309) | -0.772 (0.655) | -9.204*** (2.740) | -1.776** (0.772) | -7.450*** (0.833) | |
| *R*^2^ | 0.347 | 0.384 | 0.301 | 0.694 | 0.141 | 0.246 | 0.135 | 0.079 | 0.311 | 0.162 | 0.395 | 0.382 | 0.262 | 0.464 | |
| Observations | 3,897 | 5,826 | 508 | 114 | 9,505 | 3,047 | 489 | 16,877 | 2,915 | 5,812 | 499 | 3,598 | 1,489 | 1,067 | |

Note:

PA categories: Alto Mayo (Protection Forest), Bahuaja-Sonene (National Park), de Pui Pui (Protection Forest), de Tingo María (National Park), del Manu (National Park), del Río Abiseo (National Park), Machupicchu (Historical Sanctuary), Pacaya-Samiria (National Reserve), San Matías-San Carlos (Protection Forest), Santiago Comaina (Reserved Zone), Tabaconas-Namballe (National Sanctuary), Tambopata (National Reserve), Yanachaga-Chemillén (National Park), Yanesha (Communal Reserve). All specifications include geographic controls. The main specification uses a first-order polynomial in distance to the boundary. Standard errors are clustered at the 10 km^2^ grid level. Boundary fixed effects are not included.

Robust standard errors in parentheses

*** p<0.01, ** p<0.05, * p<0.1
