# Supplementary material for: Causal inference in conservation policy: Evaluating the effects of Protected Areas on deforestation and well-being in the Peruvian Amazon
Source: PLoS One. 2026 Oct 14;21(10):e0337468. doi: 10.1371/journal.pone.0337468 (PMC13667790; doi:10.1371/journal.pone.0337468)
Supplement: Supplementary file 4 [file pone.0337468.s003.docx]

**S3 Table. Heterogeneous Effects: Deforestation Impact by PA Associated with Each Buffer Zone**

|  | Alto Mayo | Bahuaja- Sonene | de Pui Pui | de Tingo María | del Manu | del Río Abiseo | Machupicchu | Pacaya-Samiria | San Matías-San Carlos | Santiago Comaina | Tabaconas-Namballe | Tambopata | Yanachaga-Chemillén | Yanesha | |
| --- | --- | --- | --- | --- | --- | --- | --- | --- | --- | --- | --- | --- | --- | --- | --- |
| 1 km | | | | | | | | | | | | | | |  |
| BZ | -2.956** (1.259) | -4.956*** (1.260) | 0.243 (0.673) | 2.489 (4.572) | -1.336** (0.549) | -4.521*** (1.550) | -0.00400 (1.352) | -0.252 (0.317) | -0.911 (1.650) | 0.485 (0.558) | 2.280** (0.924) | -3.237 (2.100) | -0.0278 (0.694) | -8.054*** (2.381) | |
| *R*^2^ | 0.306 | 0.477 | 0.413 | 0.485 | 0.285 | 0.325 | 0.183 | 0.068 | 0.257 | 0.195 | 0.477 | 0.338 | 0.132 | 0.191 | |
| Observations | 1,394 | 1,245 | 355 | 92 | 1,597 | 703 | 173 | 4,055 | 985 | 1,597 | 179 | 939 | 576 | 338 | |
| 5 km | | | | | | | | | | | | | | |  |
| BZ | -4.311** (1.652) | -0.539 (1.620) | -0.862 (1.095) | 1.420 (3.169) | -1.564** (0.740) | -3.375* (2.020) | -1.248* (0.703) | 0.750** (0.363) | -2.577 (1.553) | 0.224 (0.429) | 1.536 (1.206) | -2.140 (2.043) | 0.0824 (0.736) | -9.728*** (3.004) | |
| *R*^2^ | 0.291 | 0.407 | 0.490 | 0.250 | 0.248 | 0.281 | 0.183 | 0.090 | 0.274 | 0.153 | 0.377 | 0.215 | 0.103 | 0.175 | |
| Observations | 3,532 | 3,637 | 784 | 310 | 4,972 | 2,571 | 722 | 13,721 | 2,090 | 3,929 | 457 | 2,783 | 1,286 | 650 | |
| 10 km | | | | | | | | | | | | | | |  |
| BZ | -6.301*** (2.190) | -0.449 (1.610) | 0.00694 (1.205) | 3.648 (4.455) | -1.355** (0.609) | -0.416 (1.956) | -1.340* (0.732) | 0.671 (0.420) | -5.185*** (1.853) | 0.772* (0.391) | 0.774 (1.096) | -0.818 (2.751) | -1.568* (0.886) | -6.663* (3.194) | |
| *R*^2^ | 0.284 | 0.368 | 0.467 | 0.215 | 0.198 | 0.275 | 0.138 | 0.085 | 0.249 | 0.128 | 0.213 | 0.191 | 0.061 | 0.166 | |
| Observations | 5,209 | 5,364 | 1,359 | 632 | 7,318 | 4,568 | 1,169 | 20,396 | 2,983 | 5,669 | 816 | 4,101 | 1,918 | 747 | |

Note:

BZ associated with each PA: Alto Mayo (Protection Forest), Bahuaja-Sonene (National Park), de Pui Pui (Protection Forest), de Tingo María (National Park), del Manu (National Park), del Río Abiseo (National Park), Machupicchu (Historical Sanctuary), Pacaya-Samiria (National Reserve), San Matías-San Carlos (Protection Forest), Santiago Comaina (Reserved Zone), Tabaconas-Namballe (National Sanctuary), Tambopata (National Reserve), Yanachaga-Chemillén (National Park), Yanesha (Communal Reserve). All specifications include geographic controls. The main specification uses a first-order polynomial in distance to the boundary. Standard errors are clustered at the 10 km^2^ grid level. Boundary fixed effects are not included.

Robust standard errors in parentheses

*** p<0.01, ** p<0.05, * p<0.1
