# Supplementary material for: Causal inference in conservation policy: Evaluating the effects of Protected Areas on deforestation and well-being in the Peruvian Amazon
Source: PLoS One. 2026 Oct 14;21(10):e0337468. doi: 10.1371/journal.pone.0337468 (PMC13667790; doi:10.1371/journal.pone.0337468)
Supplement: Supplementary file 5 [file pone.0337468.s006.docx]

**S1. Robustness tests for the Regression Discontinuity Design**

Following Cattaneo et al. (2020), three robustness checks were conducted to assess the consistency of the estimated effects on both deforestation and household welfare.

The first test applies a donut-type RDD continuity check. Observations located within 2.5 km of the protected area boundary were incrementally excluded in 0.5 km steps, across three model specifications. The analysis was restricted to a 5 km bandwidth on either side of the boundary for both Protected Areas (PAs) and Buffer Zones (BZs). This approach tests the sensitivity of the estimates to observations closest to the boundary, which may be subject to manipulation or classification ambiguity (Cattaneo et al., 2020). It also helps assess whether the treatment-control assignment, based on whether the PA or BZ boundary intersects a given grid cell, affects the results.

The findings confirm that the estimated reductions in deforestation remain robust across all specifications, including third-degree polynomials in latitude and longitude, as well as linear and quadratic polynomials in distance to the boundary (Table 1). The effects are consistent across all bandwidths for observations within PAs. In the case of BZs, the results also suggest a reduction in deforestation; although the estimates are smaller in magnitude than for PAs, they remain statistically significant across most donut-hole radius.

Regarding welfare outcomes, previous results showed small but significant coefficients for income and expenditure near BZ boundaries. When the donut exclusion is applied, removing observations closest to the boundary, the estimates retain their expected sign and become statistically significant (Table 2). This may reflect treatment heterogeneity. Since the estimator captures the average treatment effect on the treated, grid cells located deeper within BZs are often closer to a PA, which may amplify the observed reduction in income and expenditure. As the analysis moves further from the boundary, the estimates become more consistent and significant.

Table 1. Continuity analysis of deforestation using a donut-type RDD

|  |  |  | PA | | |  | BZ | | | |
| --- | --- | --- | --- | --- | --- | --- | --- | --- | --- | --- |
| Donut-hole radius km | Distance to the boundary |  | Treatment | R2 | Observations |  | Treatment | R2 | Observations | |
| Third-order Polynomial in Latitude and Longitude | | | | | | | | | |  |
| 0.5 |  |  | -4.028*** | 0.217 | 27,272 |  | -0.963** | 0.330 | 31,924 | |
|  |  |  | (0.436) |  |  |  | (0.454) |  |  | |
| 1 |  |  | -4.184*** | 0.228 | 23,530 |  | -0.977** | 0.338 | 27,216 | |
|  |  |  | (0.454) |  |  |  | (0.482) |  |  | |
| 1.5 | 5 km |  | -4.292*** | 0.232 | 20,013 |  | -0.906* | 0.344 | 22,883 | |
|  |  |  | (0.477) |  |  |  | (0.512) |  |  | |
| 2 |  |  | -4.277*** | 0.240 | 16,712 |  | -0.847 | 0.348 | 18,869 | |
|  |  |  | (0.488) |  |  |  | (0.534) |  |  | |
| 2.5 |  |  | -4.247*** | 0.245 | 13,589 |  | -0.705 | 0.351 | 15,155 | |
|  |  |  | (0.478) |  |  |  | (0.566) |  |  | |
| Second-order Polynomial in Distance to the boundary | | | | | | | | | |  |
| 0.5 |  |  | -3.815*** | 0.213 | 27,272 |  | -1.185* | 0.312 | 31,924 | |
|  |  |  | (0.635) |  |  |  | (0.609) |  |  | |
| 1 |  |  | -4.360*** | 0.223 | 23,530 |  | -1.339* | 0.320 | 27,216 | |
|  |  |  | (0.757) |  |  |  | (0.736) |  |  | |
| 1.5 | 5 km |  | -4.921*** | 0.228 | 20,013 |  | -1.342 | 0.324 | 22,883 | |
|  |  |  | (0.914) |  |  |  | (0.885) |  |  | |
| 2 |  |  | -4.508*** | 0.235 | 16,712 |  | -1.293 | 0.327 | 18,869 | |
|  |  |  | (1.065) |  |  |  | (1.082) |  |  | |
| 2.5 |  |  | -4.632*** | 0.240 | 13,589 |  | -1.705 | 0.330 | 15,155 | |
|  |  |  | (1.160) |  |  |  | (1.288) |  |  | |
| First-order Polynomial in Distance to the boundary | | | | | | | | | |  |
| 0.5 |  |  | -3.711*** | 0.212 | 27,272 |  | -1.124* | 0.312 | 31,924 | |
|  |  |  | (0.608) |  |  |  | (0.609) |  |  | |
| 1 |  |  | -4.268*** | 0.223 | 23,530 |  | -1.288* | 0.320 | 27,216 | |
|  |  |  | (0.724) |  |  |  | (0.737) |  |  | |
| 1.5 | 5 km |  | -4.796*** | 0.227 | 20,013 |  | -1.274 | 0.324 | 22,883 | |
|  |  |  | (0.862) |  |  |  | (0.889) |  |  | |
| 2 |  |  | -4.423*** | 0.235 | 16,712 |  | -1.266 | 0.327 | 18,869 | |
|  |  |  | (1.005) |  |  |  | (1.087) |  |  | |
| 2.5 |  |  | -4.555*** | 0.240 | 13,589 |  | -1.705 | 0.330 | 15,155 | |
|  |  |  | (1.085) |  |  |  | (1.289) |  |  | |

Note: Robust standard errors in parentheses

*** p<0.01, ** p<0.05, * p<0.1

Table 2. Continuity analysis of income and expenditure using a donut-type RDD

|  |  |  | Income | | |  | Expenditure | | | |
| --- | --- | --- | --- | --- | --- | --- | --- | --- | --- | --- |
| Donut-hole radius km | Distance to the boundary |  | Treatment | R2 | Observations |  | Treatment | R2 | Observations | |
| Third- order Polynomial in Latitude and Longitude | | | | | | | | | |  |
| 0.5 |  |  | -0.209 | 0.168 | 14,602 |  | -0.077 | 0.356 | 14,602 | |
|  |  |  | (0.163) |  |  |  | (0.097) |  |  | |
| 1 |  |  | -0.292^*^ | 0.165 | 12,021 |  | -0.128 | 0.362 | 12,021 | |
|  |  |  | (0.155) |  |  |  | (0.101) |  |  | |
| 1.5 | 5 km |  | -0.381^**^ | 0.151 | 9,369 |  | -0.197^*^ | 0.356 | 9,369 | |
|  |  |  | (0.167) |  |  |  | (0.103) |  |  | |
| 2 |  |  | -0.502^***^ | 0.179 | 7,604 |  | -0.225^**^ | 0.337 | 7,604 | |
|  |  |  | (0.148) |  |  |  | (0.112) |  |  | |
| 2.5 |  |  | -0.389^*^ | 0.186 | 5,615 |  | -0.185 | 0.335 | 5,615 | |
|  |  |  | (0.202) |  |  |  | (0.152) |  |  | |
| Second-order Polynomial in Distance to the boundary | | | | | | | | | |  |
| 0.5 |  |  | -0.336 | 0.168 | 14,602 |  | -0.254 | 0.357 | 14,602 | |
|  |  |  | (0.259) |  |  |  | (0.156) |  |  | |
| 1 |  |  | -0.766^**^ | 0.165 | 12,021 |  | -0.538^**^ | 0.364 | 12,021 | |
|  |  |  | (0.362) |  |  |  | (0.239) |  |  | |
| 1.5 | 5 km |  | -0.953^**^ | 0.150 | 9,369 |  | -0.603^***^ | 0.354 | 9,369 | |
|  |  |  | (0.381) |  |  |  | (0.207) |  |  | |
| 2 |  |  | -1.113^***^ | 0.177 | 7,604 |  | -0.797^***^ | 0.335 | 7,604 | |
|  |  |  | (0.374) |  |  |  | (0.228) |  |  | |
| 2.5 |  |  | -0.596 | 0.185 | 5,615 |  | -0.593 | 0.334 | 5,615 | |
|  |  |  | (0.751) |  |  |  | (0.491) |  |  | |
| First-order Polynomial in Distance to the boundary | | | | | | | | | |  |
| 0.5 |  |  | -0.513^*^ | 0.168 | 14,602 |  | -0.376^**^ | 0.355 | 14,602 | |
|  |  |  | (0.283) |  |  |  | (0.177) |  |  | |
| 1 |  |  | -1.075^**^ | 0.164 | 12,021 |  | -0.758^***^ | 0.362 | 12,021 | |
|  |  |  | (0.417) |  |  |  | (0.281) |  |  | |
| 1.5 | 5 km |  | -1.248^***^ | 0.150 | 9,369 |  | -0.790^***^ | 0.353 | 9,369 | |
|  |  |  | (0.422) |  |  |  | (0.223) |  |  | |
| 2 |  |  | -1.366^***^ | 0.176 | 7,604 |  | -1.017^***^ | 0.334 | 7,604 | |
|  |  |  | (0.467) |  |  |  | (0.266) |  |  | |
| 2.5 |  |  | -1.113 | 0.184 | 5,615 |  | -1.023^*^ | 0.332 | 5,615 | |
|  |  |  | (0.981) |  |  |  | (0.554) |  |  | |

Note: Robust standard errors in parentheses

*** p<0.01, ** p<0.05, * p<0.1

To further assess the robustness of the results, placebo tests were conducted using bandwidths ranging from 1 to 20 km around the boundaries of PAs and BZs. These tests evaluate whether the estimated effects are sensitive to the choice of spatial window.

Consistent with previous findings, deforestation within PAs shows a gradual and significant reduction as the bandwidth increases (Fig 1A). In BZs, the reduction in forest loss remains relatively stable across bandwidths and also yields statistically significant effects (Fig 1B).

Similar patterns are observed in welfare analysis. The estimated impacts on household income (Fig 2A) and expenditure (Fig 2B) remain consistent across bandwidths, reinforcing the validity of the main results.

Fig 1. Estimated coefficients for deforestation using varying bandwidths around PA and BZ boundaries

Fig 2. Estimated coefficients for income and expenditure using varying bandwidths around BZ boundaries

To assess the validity of the discontinuity generated by the boundaries of the analyzed areas, a falsification test was conducted. This involved creating artificial boundaries slightly above and below the true discontinuity at distance zero from the protected area edge and re-estimating the treatment effects.

The results show that these artificial boundaries do not produce significant effects on deforestation control within Protected Areas (Fig 3A). In Buffer Zones, the estimates are diffuse and lack statistical significance (Fig 3B). Similarly, the falsified boundaries do not significantly affect the estimated impacts on household income (Fig 4A) or expenditure (Fig 4B) within Buffer Zones.

Fig 3. Estimated coefficients for deforestation using artificial boundaries around PAs and BZs

Fig 4. Estimated coefficients for income and expenditure using artificial boundaries around BZs
