# Supplementary material for: Causal inference in conservation policy: Evaluating the effects of Protected Areas on deforestation and well-being in the Peruvian Amazon
Source: PLoS One. 2026 Oct 14;21(10):e0337468. doi: 10.1371/journal.pone.0337468 (PMC13667790; doi:10.1371/journal.pone.0337468)
Supplement: Supplementary file 6 [file pone.0337468.s007.docx]

**S2. Robustness tests for the Staggered Difference-in-Differences Model**

As described in the main text, the parallel trends assumption was formally tested via a joint test of pre-treatment coefficients across cohorts (p-values > 0.05) and is further supported visually by the pre-treatment event-time estimates shown in each robustness check below.

The validity of short-term results was first assessed through a falsification test using alternative bandwidths. Following the same logic applied in the long-term analysis, the estimates should remain stable when the bandwidth is expanded. As shown in Fig 5, increasing the bandwidth from 5 to 10 km does not produce significant changes in the estimated effects. Across all three temporal aggregations, for PAs, BZs, and Regional Conservation Areas (RCAs), the estimates remain consistent, and the parallel trends test does not reject the null hypothesis of parallel pre-trends.

Additionally, a donut-type DiD model was conducted. Observations located within ±0.5 km and ±1 km of the boundary were excluded, using a 5 km bandwidth on either side of the PA, BZ, and RCA boundaries. The results, presented in Fig 6, confirm the previous findings: none of the three categories show significant short-term effects on deforestation. The pre-trend test confirms these results as well.

Fig 1. Falsification test with alternative bandwidths around PA, BZ, and RCA boundaries

**Fig 2. Donut-type DiD excluding observations within ±0.5 km and ±1 km of the boundary**
