# Supplementary material for: Causal inference in conservation policy: Evaluating the effects of Protected Areas on deforestation and well-being in the Peruvian Amazon
Source: PLoS One. 2026 Oct 14;21(10):e0337468. doi: 10.1371/journal.pone.0337468 (PMC13667790; doi:10.1371/journal.pone.0337468)
Supplement: Peer review history

# Peer review: Decision Letter 0

**DOI:** 10.1371/journal.pone.0337468.r001

**Authors:** Mitiku Badasa Moisa

10 Feb 2026

Dear Dr. Paredes Quiroz,

Thank you for submitting your manuscript to PLOS ONE. After careful consideration, we feel that it has merit but does not fully meet PLOS ONE’s publication criteria as it currently stands. Therefore, we invite you to submit a revised version of the manuscript that addresses the points raised during the review process.

Please submit your revised manuscript by Mar 27 2026 11:59PM. If you will need more time than this to complete your revisions, please reply to this message or contact the journal office at plosone@plos.org. When you're ready to submit your revision, log on to https://www.editorialmanager.com/pone/ and select the 'Submissions Needing Revision' folder to locate your manuscript file.

- A letter that responds to each point raised by the academic editor and reviewer(s). You should upload this letter as a separate file labeled 'Response to Reviewers'.
- A marked-up copy of your manuscript that highlights changes made to the original version. You should upload this as a separate file labeled 'Revised Manuscript with Track Changes'.
- An unmarked version of your revised paper without tracked changes. You should upload this as a separate file labeled 'Manuscript'.

If you would like to make changes to your financial disclosure, please include your updated statement in your cover letter. Guidelines for resubmitting your figure files are available below the reviewer comments at the end of this letter.

If applicable, we recommend that you deposit your laboratory protocols in protocols.io to enhance the reproducibility of your results. Protocols.io assigns your protocol its own identifier (DOI) so that it can be cited independently in the future. For instructions see: https://journals.plos.org/plosone/s/submission-guidelines#loc-laboratory-protocols. Additionally, PLOS ONE offers an option for publishing peer-reviewed Lab Protocol articles, which describe protocols hosted on protocols.io. Read more information on sharing protocols at https://plos.org/protocols?utm_medium=editorial-email&utm_source=authorletters&utm_campaign=protocols.

We look forward to receiving your revised manuscript.

Kind regards,

Mitiku Badasa Moisa

Academic Editor

PLOS One

Journal Requirements:

When submitting your revision, we need you to address these additional requirements.

1. Please ensure that your manuscript meets PLOS ONE's style requirements, including those for file naming. The PLOS ONE style templates can be found at

https://journals.plos.org/plosone/s/file?id=wjVg/PLOSOne_formatting_sample_main_body.pdf and https://journals.plos.org/plosone/s/file?id=ba62/PLOSOne_formatting_sample_title_authors_affiliations.pdf

2. When completing the data availability statement of the submission form, you indicated that you will make your data available on acceptance. We strongly recommend all authors decide on a data sharing plan before acceptance, as the process can be lengthy and hold up publication timelines. Please note that, though access restrictions are acceptable now, your entire data will need to be made freely accessible if your manuscript is accepted for publication. This policy applies to all data except where public deposition would breach compliance with the protocol approved by your research ethics board. If you are unable to adhere to our open data policy, please kindly revise your statement to explain your reasoning and we will seek the editor's input on an exemption. Please be assured that, once you have provided your new statement, the assessment of your exemption will not hold up the peer review process.

3. Please amend your authorship list in your manuscript file to include author Teresa Liliana Paredes Quiroz, Karen Ilse Eckhardt Rovalino.

4. Please amend the manuscript submission data (via Edit Submission) to include author Teresa L. Paredes and Karen I. Eckhardt.

5. We note that Figure 1 in your submission contain copyrighted images. All PLOS content is published under the Creative Commons Attribution License (CC BY 4.0), which means that the manuscript, images, and Supporting Information files will be freely available online, and any third party is permitted to access, download, copy, distribute, and use these materials in any way, even commercially, with proper attribution. For more information, see our copyright guidelines: http://journals.plos.org/plosone/s/licenses-and-copyright.

We require you to either (1) present written permission from the copyright holder to publish these figures specifically under the CC BY 4.0 license, or (2) remove the figures from your submission:

a. You may seek permission from the original copyright holder of Figure 1 to publish the content specifically under the CC BY 4.0 license.

We recommend that you contact the original copyright holder with the Content Permission Form (http://journals.plos.org/plosone/s/file?id=7c09/content-permission-form.pdf) and the following text:

“I request permission for the open-access journal PLOS ONE to publish XXX under the Creative Commons Attribution License (CCAL) CC BY 4.0 (http://creativecommons.org/licenses/by/4.0/). Please be aware that this license allows unrestricted use and distribution, even commercially, by third parties. Please reply and provide explicit written permission to publish XXX under a CC BY license and complete the attached form.”

Please upload the completed Content Permission Form or other proof of granted permissions as an "Other" file with your submission.

In the figure caption of the copyrighted figure, please include the following text: “Reprinted from [ref] under a CC BY license, with permission from [name of publisher], original copyright [original copyright year].”

b. If you are unable to obtain permission from the original copyright holder to publish these figures under the CC BY 4.0 license or if the copyright holder’s requirements are incompatible with the CC BY 4.0 license, please either i) remove the figure or ii) supply a replacement figure that complies with the CC BY 4.0 license. Please check copyright information on all replacement figures and update the figure caption with source information. If applicable, please specify in the figure caption text when a figure is similar but not identical to the original image and is therefore for illustrative purposes only.

6. We note that Figure(s) 2, 7, 8, S2 in your submission contain [map/satellite] images which may be copyrighted. All PLOS content is published under the Creative Commons Attribution License (CC BY 4.0), which means that the manuscript, images, and Supporting Information files will be freely available online, and any third party is permitted to access, download, copy, distribute, and use these materials in any way, even commercially, with proper attribution. For these reasons, we cannot publish previously copyrighted maps or satellite images created using proprietary data, such as Google software (Google Maps, Street View, and Earth). For more information, see our copyright guidelines: http://journals.plos.org/plosone/s/licenses-and-copyright.

We require you to either (1) present written permission from the copyright holder to publish these figures specifically under the CC BY 4.0 license, or (2) remove the figures from your submission:

a. You may seek permission from the original copyright holder of Figure(s) 2, 7, 8, S2 to publish the content specifically under the CC BY 4.0 license.

We recommend that you contact the original copyright holder with the Content Permission Form (http://journals.plos.org/plosone/s/file?id=7c09/content-permission-form.pdf) and the following text:

“I request permission for the open-access journal PLOS ONE to publish XXX under the Creative Commons Attribution License (CCAL) CC BY 4.0 (http://creativecommons.org/licenses/by/4.0/). Please be aware that this license allows unrestricted use and distribution, even commercially, by third parties. Please reply and provide explicit written permission to publish XXX under a CC BY license and complete the attached form.”

Please upload the completed Content Permission Form or other proof of granted permissions as an "Other" file with your submission.

In the figure caption of the copyrighted figure, please include the following text: “Reprinted from [ref] under a CC BY license, with permission from [name of publisher], original copyright [original copyright year].”

b. If you are unable to obtain permission from the original copyright holder to publish these figures under the CC BY 4.0 license or if the copyright holder’s requirements are incompatible with the CC BY 4.0 license, please either i) remove the figure or ii) supply a replacement figure that complies with the CC BY 4.0 license. Please check copyright information on all replacement figures and update the figure caption with source information. If applicable, please specify in the figure caption text when a figure is similar but not identical to the original image and is therefore for illustrative purposes only.

The following resources for replacing copyrighted map figures may be helpful:

USGS National Map Viewer (public domain): http://viewer.nationalmap.gov/viewer/

The Gateway to Astronaut Photography of Earth (public domain): http://eol.jsc.nasa.gov/sseop/clickmap/

Maps at the CIA (public domain): https://www.cia.gov/library/publications/the-world-factbook/index.html and https://www.cia.gov/library/publications/cia-maps-publications/index.html

NASA Earth Observatory (public domain): http://earthobservatory.nasa.gov/

Landsat: http://landsat.visibleearth.nasa.gov/

USGS EROS (Earth Resources Observatory and Science (EROS) Center) (public domain): http://eros.usgs.gov/#

Natural Earth (public domain): http://www.naturalearthdata.com/

7. If the reviewer comments include a recommendation to cite specific previously published works, please review and evaluate these publications to determine whether they are relevant and should be cited. There is no requirement to cite these works unless the editor has indicated otherwise.

[Note: HTML markup is below. Please do not edit.]

Reviewers' comments:

Reviewer's Responses to Questions

**Comments to the Author**

1. Is the manuscript technically sound, and do the data support the conclusions?

Reviewer #1: Partly

Reviewer #2: Yes

Reviewer #3: Partly

**********

2. Has the statistical analysis been performed appropriately and rigorously?

Reviewer #1: I Don't Know

Reviewer #2: Yes

Reviewer #3: Yes

**********

3. Have the authors made all data underlying the findings in their manuscript fully available?

The PLOS Data policy

Reviewer #1: No

Reviewer #2: Yes

Reviewer #3: Yes

**********

4. Is the manuscript presented in an intelligible fashion and written in standard English?

Reviewer #1: Yes

Reviewer #2: Yes

Reviewer #3: Yes

**********

Reviewer #1: The identification strategy requires clearer justification, particularly regarding the validity of the RDD assumptions near PA and BZ boundaries.

The distinction between short-term and long-term effects is interesting, but the temporal thresholds used to define these periods are not sufficiently explained.

Welfare impacts are reported, yet the mechanisms through which PAs and BZs affect household income and expenditure remain underdeveloped.

The negative welfare effects near indirect-use PAs raise important equity concerns, but the analysis stops short of exploring policy-relevant mitigation strategies.

The treatment of Regional Conservation Areas is exploratory and underpowered, limiting the strength of conclusions drawn for this category.

While the study contributes new empirical evidence for the Peruvian Amazon, its conceptual novelty is moderate, as similar PA impact evaluations exist in other tropical regions.

The policy implications would benefit from clearer differentiation between national PAs, BZs, and RCAs in terms of governance and management desig

Reviewer #2: The manuscript presents an interesting study in which the authors evaluate the effects of Protected Areas (PAs), their Buffer Zones (BZs), and Regional Conservation Areas (RCAs) on deforestation and household welfare from 2001 to 2023. Overall, the organization of the study is acceptable, and the methodological approach is appropriate. However, the Discussion section requires further elaboration. I recommend expanding this section and including a dedicated subsection on study limitations, particularly highlighting data constraints and other factors that may have influenced the analysis. In addition, the Discussion currently relies heavily on local and regional literature; the authors are encouraged to incorporate more recent and relevant global literature on the topic.

Specific comments are provided below:

Line 19: Revise to “Study area” only; there is no need to mention “units of analysis” at this point.

Lines 236–238: This content pertains to the methods/analysis and should be relocated to the appropriate section rather than the Results section.

Please expand the titles of all figures and tables throughout the manuscript. The current titles are too brief and do not provide sufficient information to fully understand the content presented.

Reviewer #3: The manuscript addresses an important and policy-relevant question and applies appropriate causal inference methods by assessing the impact of PAs, BZs and to regional conservation areas RCAs in the Peruvian Amazon using RDD and modern staggered DiD methods. However, revisions are required to more clearly state the study’s novelty, strengthen the discussion of key causal assumptions and welfare implications, and clarify the interpretation findings related to RCAs.

1- The introduction could be strengthened by briefly referencing how buffer zones and RCAs compare to other protected area types in the existing literature, even if only conceptually.

2- In several instances (e.g., “however, the literature cautions against evaluating these targets solely by the extent of protected land (7–9)” and the discussion of studies questioning the real impact of PAs), the cited literature is referenced in a collective manner. The manuscript would benefit from a more explicit discussion of these studies, including a brief explanation of their key findings and how they directly support the stated claims, rather than listing multiple references without differentiation.

3- The assumption of the (RDD) model, that unobserved variables do not change discontinuously at the boundary needs stronger justification.

4- justify the choice of a 1 km² grid resolution and discuss potential treatment misclassification arising from partial overlaps with PA or BZ boundaries.

5- In the sentence, leaving an empirical gap that this study addresses by extending the temporal horizon and territorial scope of the analysis, is a strong claim. Could you add more brief explanations in three sentences, four, such as, data limitations, methodological challenges, etc.

6- The description of control variables in the text is concise, but without a sufficiently detailed Table S1 and 2, the variables are not easily interpretable.

Technical Comments:

1- There is inconsistency in the citation format throughout the manuscript (e.g., use of square brackets versus parentheses). Grupp et al.,

The reference style should be standardized according to the journal’s guidelines.

2- The images are not well-organized; it is recommended to arrange them neatly within the text. Also, need higher resolution.

3- Minor language edits are needed, including inconsistent capitalization.

4- Figure captions appear embedded within the main text rather than being placed directly below the figures.

5- Some sentences are long and slightly overloaded and combined different ideas at once, e.g., In the Peruvian case, studies have primarily focused on the impacts of PAs on deforestation, often overlooking BZs and RCAs. Díaz y Miranda (30), Miranda et al. (31), and Orihuela and Pérez (32) report significant reductions in deforestation, although with limited or even negative effects on household welfare.

6- Key robustness results should be reported directly in the manuscript or formal supplementary tables (e.g., S1–S2), rather than through external Word documents. Also, the claim that the estimates satisfy the parallel trends assumption should be supported by more explanation.

7- Regression models should be numbered for clarity and ease of reference.

8- The term Donut-hole radio should be corrected to Donut-hole radius.

**********

what does this mean?). If published, this will include your full peer review and any attached files.

If you choose “no”, your identity will remain anonymous but your review may still be made public.

**Do you want your identity to be public for this peer review?**  For information about this choice, including consent withdrawal, please see our Privacy Policy

Reviewer #1: No

Reviewer #2: No

Reviewer #3: **Yes:**  Lamia Alyami

**********

[NOTE: If reviewer comments were submitted as an attachment file, they will be attached to this email and accessible via the submission site. Please log into your account, locate the manuscript record, and check for the action link "View Attachments". If this link does not appear, there are no attachment files.]

To ensure your figures meet our technical requirements, please review our figure guidelines: https://journals.plos.org/plosone/s/figures

You may also use PLOS’s free figure tool, NAAS, to help you prepare publication quality figures: https://journals.plos.org/plosone/s/figures#loc-tools-for-figure-preparation.

NAAS will assess whether your figures meet our technical requirements by comparing each figure against our figure specifications.

# Author comment: Author response to Decision Letter 1

**DOI:** 10.1371/journal.pone.0337468.r002

2 Sep 2026

Response to Academic Editor

JR-1: Ensure the manuscript meets PLOS ONE's style requirements, including file naming conventions.

- The manuscript has been reformatted to comply with PLOS ONE’s style requirements.

JR-2: Decide on a data sharing plan before acceptance. All data must be freely accessible upon publication (except where restricted by an ethics board). If open data policy cannot be met, revise the Data

Availability Statement with justification and request an exemption.

- We have completed the data sharing plan. All processed data and analysis scripts that underlie the study’s findings are publicly accessible on Zenodo at https://doi.org/10.5281/zenodo.22244470, including all processed datasets required to reproduce the models. Raw source data are publicly available via their respective institutional portals, as described in S1 Table; raw ENAHO microdata are not redistributed directly but can be downloaded on demand from INEI’s official microdata portal using the provided download script. The Data Availability Statement has been updated to reflect these provisions.

JR-3: Amend the authorship list in the manuscript file to include: Teresa Liliana Paredes Quiroz and Karen Ilse Eckhardt Rovalino.

- The authorship list in the manuscript file has been amended to include the full legal names.

JR-4: Amend the manuscript submission data (via Edit Submission) to include: Teresa L. Paredes and Karen I. Eckhardt.

- The manuscript submission metadata has been updated to include Teresa L. Paredes and Karen I. Eckhardt.

JR-5: Figure 1 contains copyrighted images. PLOS ONE requires all content to be published under CC BY 4.0. Either: (a) obtain written permission from the copyright holder to publish under CC BY 4.0 using the Content Permission Form, or (b) remove or replace the figure.

- Figure 1 is an original schematic diagram created entirely by the authors to illustrate the treatment/control zone assignment used in the RDD design, as indicated in the figure caption (“Authors elaboration”). It does not reproduce or derive from any copyrighted, proprietary, or third-party map, image, or dataset. Therefore, no permission request or Content Permission Form applies to this figure.

JR-6: Figures 2, 7, 8, and S2 contain map/satellite images that may be copyrighted (e.g., Google Maps, Street View, Earth). Either: (a) obtain permission from the copyright holder using the Content Permission Form, or (b) remove or replace the figures with public domain sources.

- We have obtained written permission from relevant copyright holders (MINAM, SERNANP, Instituto del Bien Común, SERFOR, INGEMMET, PerúPetro, MTC and IGN) to publish Figures 2, 7, 8, and S2 Fig under CC BY 4.0 license. The corresponding permission forms and official written authorizations (in Spanish and English) are included as supporting files with this submission. The figure captions have been updated to credit each institutional source accordingly.

JR-7: If reviewers recommend citing specific previously published works, review and evaluate those publications to determine whether they are relevant and should be cited. There is no requirement to cite them unless the editor indicates otherwise.

- The reviewers did not recommend specific published works to cite. We have nonetheless updated and expanded the literature discussed throughout the manuscript, particularly with additional recent global references, as detailed in our responses to Reviewer #2 and Reviewer #3 below.

Response to Reviewer 1

R1-1: The identification strategy requires clearer justification, particularly regarding the validity of the RDD assumptions near PA and BZ boundaries.

- We have strengthened the justification of the RDD identification strategy in three locations of the manuscript:

- (a) In the Empirical Strategy section, we expanded the paragraph describing the RDD assumption (lines 263- 277) to explain why it is plausible in this specific context: PA and BZ boundaries in Peru are defined through administrative and legal instruments (Law N° 26834) rather than pre-existing socioeconomic gradients; the model includes comprehensive geographic and accessibility controls; boundary fixed effects capture area-specific unobservables; and the design follows previous geographic RDD applications in Colombia and Peru where the same assumption has been argued to hold

- (b) Following the description of the running variable (lines 251-258), we added a paragraph clarifying that the use of distance to the boundary as the running variable is appropriate because PA and BZ boundaries were not drawn based on proximity to populated areas or pre-existing land-use intensity, making the cutoff plausibly exogenous to the outcomes of interest.

- (c) In the Results section, we expanded the robustness checks paragraph (lines 473-481) to explicitly connect the donut RDD, placebo tests, and artificial boundary tests to the validation of the core RDD assumption, noting that the absence of effects at false boundaries confirms that covariates do not change discontinuously at the true legal boundary

R1-2: The distinction between short-term and long-term effects is interesting, but the temporal thresholds used to define these periods are not sufficiently explained.

- We have clarified the temporal thresholds underlying the long-term/short-term distinction in three locations of the manuscript:

- (a) In the Introduction (lines 142-152), we added a brief explanation indicating that the 2001 threshold is driven by data availability, specifically, the onset of high-resolution annual deforestation records, and directing the reader to the Empirical Strategy section for the full technical justification

- (b) At the end of the Empirical Strategy section (lines 337-351), we replaced the brief closing remark with a detailed explanation of: why areas established before 2001 cannot be evaluated with a panel design; the substantive meaning of long-term (minimum 22 years of accumulated protection by 2023); why the DiD sample is restricted to 2006-2018 (minimum five pre- and post-treatment years); and the clarification that the temporal labels do not imply a methodological hierarchy between the RDD and DiD

- (c) In the Short-term DiD subsection (lines 487-492), we expanded the sentence describing the sample window to explicitly justify both bounds: the lower bound of 2006 (minimum pre-treatment window to test parallel trends) and the upper bound of 2018 (minimum five post-treatment years for effects to materialize)

R1-3: Welfare impacts are reported, yet the mechanisms through which PAs and BZs affect household income and expenditure remain underdeveloped.

- We have expanded the welfare discussion in the Discussion section (lines 651-666) to articulate the mechanisms through which PAs and BZs affect household income and expenditure. Specifically, we identify three plausible channels: (a) regulatory restrictions on resource extraction and agricultural expansion established in PA Master Plans, which are more stringent near indirect-use PAs and directly limit household income sources; (b) geographic remoteness and limited market access in BZ areas, which constrains the ability of households to substitute restricted activities; and (c) the uneven distribution of income from compatible activities such as ecotourism, which does not systematically benefit BZ households. We also clarify that the observed welfare costs likely reflect the absence of compensatory policies rather than conservation itself and retain the caveat that income and expenditure are partial welfare measures

R1-4: The negative welfare effects near indirect-use PAs raise important equity concerns, but the analysis stops short of exploring policy-relevant mitigation strategies.

- We have addressed this comment in two ways. First, the welfare paragraph in the Discussion (lines 667-671) was extended to flag explicitly the equity concerns specific to indirect-use PAs. Second, a dedicated “Policy Implications” sections was created after Study Limitations, consolidating and reorganizing policy-relevant content that was previously dispersed across the Discussion and embedded within Study Limitations. The new section identifies three targeted areas of intervention: compensation mechanisms including PES, REDD+, and MERESE schemes, supported by [36] Morgans et al (2024), who showed for Indonesia that combining conservation restrictions with livelihood support simultaneously reduces deforestation and improves household welfare, conservation agreements and co-management frameworks adapted to local governance structures; and strengthening SINANPE’s operational capacity alongside multidimensional welfare indicators. Gender equity is explicitly identified as a design criterion across all three.

R1-5: The treatment of Regional Conservation Areas (RCAs) is exploratory and underpowered, limiting the strength of conclusions drawn for this category.

- We acknowledge this limitation and have addressed it in three locations. First, in the Empirical Strategy section (lines 318–323), we state explicitly that the RCA analysis is exploratory due to the small sample size of 9 RCAs and the accompanying limitations in statistical power. Second, in the Results section (lines 524-536), we broaden the RCA discussion to indicate that non-significance arises from data limitations rather than ineffectiveness and note that the estimated directions align with a conservation effect. We situate the RCA category within the broader literature on subnational conservation governance, drawing on [54] Qin et al. (2024) and [55] Virtanen et al. (2025). Third, in the Study Limitations subsection (lines 742-748), we include a dedicated point on the limited statistical power of the RCA analysis and advocate for future research employing larger samples and longer post-treatment windows

R1-6: The study's conceptual novelty is moderate, as similar PA impact evaluations exist in other tropical regions.

- We acknowledge the existence of impact evaluations of PAs in tropical regions and thus recognize that the study’s conceptual contribution is contextual rather than foundational. The final paragraph of the Introduction (lines 157-165) has been revised to explicitly acknowledge this while detailing aspects unique to the Peruvian context: (i) the inaugural application of the staggered DiD framework to PA evaluation in Peru; (ii) the joint treatment of three governance categories within a single causal-inference framework; (iii) the longest temporal window present in the national literature; and (iv) the simultaneous estimation of ecological and socioeconomic outcomes under a common identification strategy. Rather than asserting broad conceptual originality, the study repositions its contribution at the nexus of methodological rigor and contextual specificity

R1-7: The policy implications would benefit from clearer differentiation between national PAs, BZs, and RCAs in terms of governance and management design.

- The Policy Implications section has been reorganized and rewritten to derive differentiated recommendations directly from the heterogeneous outcomes observed for each governance category. For nationally administered PAs, recommendations are anchored in results by PA type: the substantial performance of Communal Reserves and Protection Forests under active pressure (up to 9.0 and 6.8 ha/km2, respectively) contrasts with the lack of effects in large remote areas such as Pacaya Samiria and Manu, indicating that investments in management should be prioritized where external pressures are demonstrably high. For BZs, policy guidance addresses the dual challenge identified in the results: effective long-term deforestation containment alongside concentrated welfare costs near indirect-use PAs, where income losses reach as much as 1.2 times the poverty line. For RCAs, recommendations emphasize the institutional preconditions necessary for effectiveness, drawing on [79] Cano et al. (2026) regarding the Maijuna-Kichwa RCA. A concluding paragraph delineates cross-cutting priorities that apply across all three categories.

Response to Reviewer 2

R2-1: The Discussion section requires further elaboration. A dedicated subsection on study limitations should be included, particularly highlighting data constraints and other factors that may have influenced the analysis.

- We have reorganized and expanded the content limitations in the Discussion section by creating a dedicated subsection titled "Study limitations" (lines 711-748), which replaces the two previously dispersed limitation paragraphs. The subsection systematically addresses seven categories of limitations: (a) data constraints related to ENAHO's sampling design and its inability to capture conservation-compatible income sources; (b) potential treatment misclassification arising from the use of 1 km² grid cells with partial boundary overlaps; (c) the temporal gap affecting areas established between 2001 and 2005, which could not be included in either analysis; (d) the assumptions underlying both causal inference methods and their potential limitations in highly heterogeneous PA contexts; (e) the restriction of deforestation as the sole effectiveness metric, noting that inaccessibility and illegal extraction may confound results; (f) institutional constraints that independently affect conservation outcomes beyond what the models can estimate; and (g) the exploratory nature of the RCA analysis, as detailed in our response to Comment R1-5.

R2-2: The Discussion currently relies heavily on local and regional literature. The authors are encouraged to incorporate more recent and relevant global literature on the topic.

- We have incorporated additional recent and global literature throughout the manuscript, including: [8] Visconti et al. (2019), [9] Geldmann (2026), [54] Qin et al. (2024), [55] Virtanen et al. (2025), [56] Ramiandrisoa et al. (2026), [67] He et al. (2025), [68] den Braber et al. (2024), [69] Gaglia Alves et al. (2025), [78] Sarmiento Barletti and Rolando (2024).

R2-3: Line 19: Revise to "Study area" only; there is no need to mention "units of analysis" at this point.

- The heading of the corresponding subsection in Materials and Methods (line 170) has been revised from "Study area and units of analysis" to "Study area", as recommended. The description of the units of analysis remains in the body of the section where it is introduced in context

R2-4: Lines 236-238: This content pertains to methods/analysis and should be relocated to the appropriate section rather than the Results section.

- The description of the local conditional means test has been relocated from the Results section to the Long-term analysis, RDD subsection within Empirical Strategy, where it now appears as a methodological step preceding the model specification (lines 278-280).

R2-5: Expand the titles of all figures and tables throughout the manuscript. The current titles are too brief and do not provide sufficient information to fully understand the content presented.

- The titles of all figures and tables throughout the manuscript have been revised to be more informative. In addition, detailed captions have been added to all figures and tables.

Response to Reviewer 3

R3-1: The introduction could be strengthened by briefly referencing how buffer zones and RCAs compare to other protected area types in the existing literature, even if only conceptually.

- We have strengthened the Introduction by incorporating a paragraph (lines 92-98) that briefly positions BZs and RCAs within the broader categories examined in the extant global literature. Conceptually, BZs correspond to the multi-use or transition zones described around strictly protected areas in other regions, where permitted activities are more flexible than in the core area yet constrained by conservation objectives. RCAs are positioned alongside subnational or regionally governed reserves found in decentralized conservation systems, where effectiveness is often linked to local governance capacity and proximity to communities. The comparison remains at a conceptual level, in accordance with the reviewer’s suggestion, and does not conflate the specific legal and institutional characteristics of Peruvian categories with analogous arrangements elsewhere.

R3-2: In several instances, the cited literature is referenced in a collective manner without differentiation. The manuscript would benefit from a more explicit discussion of these studies, including a brief explanation of their key findings and how they directly support the stated claims.

- We have revised the relevant passages throughout the manuscript to differentiate previously block-cited references, replacing collective citations with individual discussions of each study’s specific findings and how they directly support the corresponding claims. This includes the following sections: lines 54-91; lines 557-569; lines 571-585; lines 614-635; lines 696-676;

R3-3: The assumption of the RDD model, that unobserved variables do not change discontinuously at the boundary, needs stronger justification.

- This concern is addressed by the revisions made in response to Reviewer 1, comment 1 (R1-1)

R3-4: Justify the choice of a 1 km² grid resolution and discuss potential treatment misclassification arising from partial overlaps with PA or BZ boundaries.

- We have addressed this comment in two locations. First, in the Study area subsection (lines 174-180), we justified the choice of 1 km² grid resolution, the source data from MINAM are available at 30 m² but processing the full Amazonian coverage at that granularity would be computationally prohibitive and clarified the treatment assignment rule following [28] Orihuela and Pérez (2019). A cell is classified as treated if the PA or BZ boundary intersects it, with distance assigned as negative for treated and positive for control cells. Second, in the Study limitations subsection (lines 719-725), we discuss the potential for treatment misclassification arising from cells that straddle the boundary, noting that the donut-type RDD robustness checks (S1 Text, Tables 1-2) confirm this does not affect the main results.

R3-5: The claim "leaving an empirical gap that this study addresses by extending the temporal horizon and territorial scope of the analysis" is a strong claim. Add approximately 3-4 sentences explaining the reasons for this gap (data limitations, methodological challenges, etc.).

- We expanded the empirical gap claim (lines 113-123) with three sentences explaining why it persisted prior to this study: (a) evaluating recently established PAs requires sufficient pre- and post-treatment data, a condition only met as the deforestation record extended further beyond their establishment dates; (b) prior Peruvian research relied on matching methods, which cannot exploit temporal variation or estimate dynamic treatment effects for areas with staggered establishment dates; and (c) Regional Conservation Areas, governed outside the SINANPE framework, have been systematically excluded from the empirical literature despite their growing role in Amazonian conservation governance

R3-6: The description of control variables in the text is concise, but without a sufficiently detailed Table S1 and S2, the variables are not easily interpretable.

- In S1 Table, we added a footnote specifying the exact set of controls used in each model. In S2 and S3 Tables, we added a footnote identifying the conservation category of each PA, which is necessary to interpret the heterogeneous effects in conjunction with Tables 2-4 of the main manuscript

R3-7: There is inconsistency in the citation format throughout the manuscript (e.g., use of square brackets versus parentheses). The reference style should be standardized according to the journal's guidelines.

- The citation format has been standardized throughout the manuscript to use square brackets consistently.

R3-8: The images are not well-organized; it is recommended to arrange them neatly within the text. Images also need higher resolution.

- Figures have been updated to higher resolution and reorganized within the manuscript to appear immediately following their first mention in the text, in accordance with PLOS ONE formatting guidelines.

R3-9: Minor language edits are needed, including inconsistent capitalization throughout the manuscript.

- We conducted a full language and style review of the manuscript, correcting inconsistent capitalization and other language inconsistencies throughout.

R3-10: Figure captions appear embedded within the main text rather than being placed directly below the figures.

- Figure and table captions have been reformatted with bold identifiers and are presented clearly separated paragraphs immediately following their first citation in the text.

R3-11: Some sentences are long and slightly overloaded, combining different ideas at once. These should be split or simplified.

- We have conducted a full style review to shorten and clarify long or overloaded sentences throughout the manuscript.

R3-12: Key robustness results should be reported directly in the manuscript or in formal supplementary tables (e.g., S1-S2), rather than through external Word documents. Additionally, the claim that the estimates satisfy the parallel trends assumption should be supported by more explanation.

- We have addressed both points. First, the key robustness results for both the RDD and DiD analyses are now reported directly in the manuscript, with tables and figures retained in the formal supplementary materials (S1 Text and S2 Text). For the RDD, the three robustness checks, donut-type RDD, placebo bandwidth tests, and boundary falsification, are now described with their main findings in the Results section (lines 473-481). For the DiD, the bandwidth falsification test and donut-type DiD are similarly described with results in the text (lines 544-552). Second, we expanded the description of the parallel trends test (lines 499-503) to explain that pre-treatment coefficients were jointly tested for each cohort, that no statistically significant violations were found across PAs, BZs, or RCAs, and that event-time plots further confirm the absence of systematic pre-trends.

R3-13: Regression models should be numbered for clarity and ease of reference.

- Both regression specifications in the Empirical Strategy section have been numbered to improve clarity. The RDD specification is designated as Equation 1, while the staggered DiD specification is designated as Equation 2.

R3-14: The term "Donut-hole radio" should be corrected to "Donut-hole radius".

- The term “Donut-hole-radio” has been corrected to “Donut-hole-radius” throughout both the manuscript and the supplementary materials.

Attachment Submitted filename: Response_to_Reviewers.pdf

# Peer review: Decision Letter 1

**DOI:** 10.1371/journal.pone.0337468.r003

**Authors:** Mitiku Badasa Moisa

30 Sep 2026

Causal inference in conservation policy: Evaluating the effects of Protected Areas on deforestation and well-being in the Peruvian Amazon

PONE-D-25-59578R1

Dear Dr. Paredes Quiroz,

We’re pleased to inform you that your manuscript has been judged scientifically suitable for publication and will be formally accepted for publication once it meets all outstanding technical requirements.

Within one week, you’ll receive an e-mail detailing the required amendments. When these have been addressed, you’ll receive a formal acceptance letter and your manuscript will be scheduled for publication.

An invoice will be generated when your article is formally accepted. Please note, if your institution has a publishing partnership with PLOS and your article meets the relevant criteria, all or part of your publication costs will be covered. Please make sure your user information is up-to-date by logging into Editorial Manager at Editorial Manager® and clicking the ‘Update My Information' link at the top of the page. For questions related to billing, please contact billing support.

If your institution or institutions have a press office, please notify them about your upcoming paper to help maximize its impact. If they’ll be preparing press materials, please inform our press team as soon as possible -- no later than 48 hours after receiving the formal acceptance. Your manuscript will remain under strict press embargo until 2 pm Eastern Time on the date of publication. For more information, please contact onepress@plos.org.

Kind regards,

Mitiku Badasa Moisa

Academic Editor

PLOS One

Additional Editor Comments (optional):

I am pleased to inform you that your paper is accepted for publication

Reviewers' comments:

Reviewer's Responses to Questions

**Comments to the Author**

Reviewer #3: All comments have been addressed

**********

2. Is the manuscript technically sound, and do the data support the conclusions?

Reviewer #3: Yes

**********

3. Has the statistical analysis been performed appropriately and rigorously?

Reviewer #3: Yes

**********

4. Have the authors made all data underlying the findings in their manuscript fully available?

The PLOS Data policy

Reviewer #3: Yes

**********

5. Is the manuscript presented in an intelligible fashion and written in standard English?

Reviewer #3: Yes

**********

Reviewer #3: (No Response)

**********

what does this mean?). If published, this will include your full peer review and any attached files.

If you choose “no”, your identity will remain anonymous but your review may still be made public.

**Do you want your identity to be public for this peer review?**  For information about this choice, including consent withdrawal, please see our Privacy Policy

Reviewer #3: No

**********

# Editor report: Acceptance letter

**DOI:** 10.1371/journal.pone.0337468.r004

**Authors:** Mitiku Badasa Moisa

PONE-D-25-59578R1

PLOS One

Dear Dr. Paredes,

I'm pleased to inform you that your manuscript has been deemed suitable for publication in PLOS One. Congratulations! Your manuscript is now being handed over to our production team.

At this stage, our production department will prepare your paper for publication. This includes ensuring the following:

* All references, tables, and figures are properly cited

* All relevant supporting information is included in the manuscript submission,

* There are no issues that prevent the paper from being properly typeset

You will receive further instructions from the production team, including instructions on how to review your proof when it is ready. Please keep in mind that we are working through a large volume of accepted articles, so please give us a few days to review your paper and let you know the next and final steps.

Lastly, if your institution or institutions have a press office, please let them know about your upcoming paper now to help maximize its impact. If they'll be preparing press materials, please inform our press team within the next 48 hours. Your manuscript will remain under strict press embargo until 2 pm Eastern Time on the date of publication. For more information, please contact onepress@plos.org.

You will receive an invoice from PLOS for your publication fee after your manuscript has reached the completed accept phase. If you receive an email requesting payment before acceptance or for any other service, this may be a phishing scheme. Learn how to identify phishing emails and protect your accounts at https://explore.plos.org/phishing.

If we can help with anything else, please email us at customercare@plos.org.

Thank you for submitting your work to PLOS One and supporting open access.

Kind regards,

PLOS One Editorial Office Staff

on behalf of

Dr. Mitiku Badasa Moisa

Academic Editor

PLOS One
